# Molecular Mechanisms of *Cassia fistula* against Epithelial Ovarian Cancer Using Network Pharmacology and Molecular Docking Approaches

**DOI:** 10.3390/pharmaceutics14091970

**Published:** 2022-09-19

**Authors:** Aqsa Kanwal, Farrukh Azeem, Habibullah Nadeem, Usman Ali Ashfaq, Rana Muhammad Aadil, A. K. M. Humayun Kober, Muhammad Shahid Riaz Rajoka, Ijaz Rasul

**Affiliations:** 1 Department of Bioinformatics and Biotechnology, Government College University Faisalabad, Faisalabad 38000, Pakistan; 2 National Institute of Food Science and Technology, University of Agriculture Faisalabad, Faisalabad 38000, Pakistan; 3 Department of Dairy and Poultry Science, Chittagong Veterinary and Animal Sciences University, Chittagong 4225, Bangladesh; 4 Laboratory of Animal Food Function, Graduate School of Agricultural Science, Tohoku University, Sendai 980-8572, Japan

**Keywords:** epithelial ovarian cancer, *Cassia fistula*, anticarcinogenic, network pharmacology, active constituents, gene ontology, molecular docking

## Abstract

Epithelial ovarian cancer (EOC) is one of the deadliest reproductive tract malignancies that form on the external tissue covering of an ovary. *Cassia fistula* is popular for its anti-inflammatory and anticarcinogenic properties in conventional medications. Nevertheless, its molecular mechanisms are still unclear. The current study evaluated the potential of *C. fistula* for the treatment of EOC using network pharmacology approach integrated with molecular docking. Eight active constituents of *C. fistula* were obtained from two independent databases and the literature, and their targets were retrieved from the SwissTargetPrediction. In total, 1077 EOC associated genes were retrieved from DisGeNET and GeneCardsSuite databases, and 800 potential targets of eight active constituents of *C. fistula* were mapped to the 1077 EOC targets and intersected targets from two databases. Ultimately, 98 potential targets were found from *C. fistula* for EOC. Finally, the protein–protein interaction network (PPI) topological interpretation revealed AKT1, CTNNB1, ESR1, and CASP3 as key targets. This is the first time four genes have been found against EOC from *C. fistula*. The major enriched pathways of these candidate genes were established by Gene Ontology (GO) and Kyoto Encyclopedia of Genes and Genomes (KEGG) investigations. To confirm the network pharmacology findings, the molecular docking approach demonstrated that active molecules have higher affinity for binding to putative targets for EOC suppression. More pharmacological and clinical research is required for the development of a drug to treat EOC.

## 1. Introduction

Ovarian cancer (OC) is the world’s seventh highest prevalent lethal gynecological malignancy and accounted for 2.5% of new cancers in women. In 2019, the number of new cases was predicted to be 222,240, while the death toll was estimated to be 14,170 [1]. The most frequent type of OC is epithelial ovarian cancer (EOC), having a 45.6% survival rate [2]. EOC originates from the ovaries, ovarian surface epithelial (OSE), oviduct, and pelvic epithelium locales. About 75% individuals are examined at later phases because EOC symptoms are non-specific and typically include abdominal bloating, distention, early satiety, nausea, changes in bowel function, urinary tract problems, back pain, fatigue, and weight loss [3]. EOCs are listed at different stages based on conformation of the cancer. The malignancy is restricted to either one or both ovaries at phase I. In phase II, the tumor also extends to the uterus and other epithelial tissues in the pelvic region. In phase III, the tumor advances at lymphoid tissue and is confined to the abdominopelvic cavity. In phase IV, both or one of the ovaries are implicated in remote metastases [4]. EOC is not a single disease, while studies show that it is composed of tumors categorized on the basis of molecular genetics. EOC composed of subtypes on the basis of closely resembled tissues encompasses serous, mucinous, endometriosis, clear-cell and transitional cell types. EOC is treated surgically, followed by chemotherapy [4]. The etiology of EOC includes stress-induced recurrent ovulation [5,6], elevated levels of estrogen [7], high levels of androgens [7], and stromal hyperactivity [8]. However, the etiology and pathogenesis of EOC is very complex and not yet fully understood.

The use of plants and their components to control different diseases in humans has an age-old history. Several therapeutic plants have been demonstrated to be effective in the administration of quality of life through antioxidant, anti-inflammation, and antidiabetic biological effects [9,10,11]. These herbal medicines, composed of hundreds of phytochemicals, are derived from many herbs, though their effects and molecular mechanisms are not well known. Network pharmacology, a new drug development technique, was introduced in Nature Biotechnology by Hopkins in 2007, updated to the present day “one-target-on-one-drug” approach to the advanced “network targets, multiple-constituent” technique [12]. Network pharmacology analyzes the complex biological mechanisms and selects specific signaling nodes to develop multi-target drug molecules. It highlights the active constituents, improves therapeutic effects of drugs, reduces toxicity and maps active constituents to disease gene to seek potential targets to develop PPI network and carry out gene annotation analysis [13,14]. It focuses to demonstrate the interaction of active constituents, potential targets, and disease-related genes by constructing active constituent/potential target signaling pathway networks [15]. In this way, it shows how the screened potential targets work to cure a disease.

*C. fistula* belongs to the subfamily Caesalpinioideae of the legume family, Fabaceae popular as Amaltas. *C. fistula* is indigenous to the Indian subcontinent, Mauritius, Thailand, China, Brazil, Sri Lanka, and southern Pakistan. It plays a central role in disease prevention because of its valuable constituents such as flavonoids, anthraquinone, chromones, coumarins, alkaloids, phytosterols, long-chain hydrocarbons, phenolic, and triterpenes. Former research confirmed that *C. fistula* showed roles in antifertility [16], antimicrobial [17], hepatoprotective [18], improved tissue regeneration and wound healing activities [19,20]. *C. fistula* also played a wide range of activities including hypoglycemic activities [21,22], antipyretic [23], larvicidal [24], anti-inflammatory [25], antioxidant and antitumor activities [26]. *C. fistula* is prescribed against epidermis, hepatic, and lung problems as well as hematemesis, chronic itchy skin, hypopigmentation, and diabetes mellitus [27]. The exact activities of its molecular mechanisms to prevent a disease are yet entirely unknown. In this investigation, a *C. fistula*-target EOC network is constructed, from analysis of compound to the interaction of potential targets, integrated with pathway analysis to investigate the molecular mechanism of *C. fistula* in order to treat EOC.

## 2. Materials and Methods

### 2.1. Phytochemical Library Construction

Phyto-constituents of *C. fistula* were acquired from the literature [28,29], KNApSAcK Family Core System database [30] (http://www.knapsackfamily.com/, accessed on 8 August 2022) and Traditional Chinese Medicine System Pharmacology (TCMSP) (https://tcmsp-e.com/tcmsp.php, accessed on 8 August 2022), which was performed by using the plant name *Cassia fistula* as a search term in kNAPSAcK database [31] and literature mining performed via Google Scholar and PubMed. Their 2D structures in .sdf file format were collected through PubChem database (https://pubchem.ncbi.nlm.nih.gov/, accessed on 10 August 2022) to construct phytochemical library. Meanwhile, their SMILES were also collected in order to investigate the pharmacodynamics attributes. Active constituents of *C. fistula* were obtained from admetSAR (http://lmmd.ecust.edu.cn/admetsar2, accessed on 13 August 2022) and TCMSP database, i.e., OB and DL greater than 30% and 0.18, respectively [32]. OB stands for oral bioavailability of pharmacological components, while DL stands for drug-component similarity, which might suggest a prospective drug.

### 2.2. Drug Target Profiles for C. fistula

SwissTargetPrediction tool (http://www.swisstargetprediction.ch/, accessed on 15 August 2022) was used to find out prospective genes targeting active compounds via providing the SMILES, while *Homo sapiens* were selected as the species. SwissTargetPrediction is extensively used program for reverse screening of chemical compounds and predicting the bioactivity of the chemicals. Protein IDs were aligned with UniProtKB (https://www.uniprot.org/help/uniprotkb, accessed on 16 August 2022) [33], performed in order to eliminate duplication.

### 2.3. Candidate Targets of C. fistula for EOC

EOC-related Human targets were retrieved from DisGeNET (https://www.disgenet.org/, accessed on 20 August 2022) and GeneCards (https://www.genecards.org/, accessed on 20 August 2022) using keyword “epithelial ovarian cancer” [34,35]. A Venn illustration was built to map drug-targets profiles of active constituents of *C. fistula* to the EOC-related targets [36] in order to obtain prospective genes of *C. fistula* to treat EOC. These mapped genes were chosen to advance the investigations.

### 2.4. Compound-Target Network

The network of active constituents of *C. fistula* with associated genes was built and analyzed through Cytoscape v3.8.2 [37]. Cytoscape is a freely available bioinformatics platform to visualize, incorporate complex networks from different types of information. Nodes of network formed represent the phyto-constituents and genes. The edges depict the interrelationship between them. A plug-in “Network Analyzer” was employed to assess the network’s topology [38]. The network was evaluated based on “degree” [39].

### 2.5. Protein–Protein Interaction (PPI) Network

To evaluate the interaction between the prospective genes of *C. fistula,* these were imported into STRING v11.5 database (https://string-db.org/, accessed on 25 August 2022) to construct a PPI network [40]. The multiple protein option was taken and potential targets were added using the “Homo sapiens” as the target species. The network was built with a confidence of 0.4. This network was further analyzed using Cytoscape for visualization and topological analysis [37]. A plug-in “CytoHubba” was used to obtain targets of higher degree. In fact, higher the degree means they are linked more [41]. “Network Analyzer” was utilized to analyze the topological properties [38].

### 2.6. Gene Functional Annotation

The gene and pathway enrichment investigation was performed through DAVID (https://david.ncifcrf.gov/home.jsp, accessed on 26 August 2022). DAVID is a database of functional annotations that can be accessed online that helps researchers comprehends the biological meanings of a huge number of genes. The gene enrichment analysis categorized the gene functions that incorporate Biological Process (BP), Cellular Component (CC) and Molecular Function (MF). Moreover, enriched pathways were filtered out through KEGG analysis [42]. The potential genes were copied into DAVID, selecting the species as the *Homo sapiens*. The probability value was set to *p* < 0.05 to select enriched pathways. To illustrate the GO annotation and KEGG pathways, bubble plots were created in R via ggplot2 package.

The highest 20 enriched pathways were chosen to construct the pathway–target network, which was constructed and examined by using Cytoscape v3.8.2 to understand interactions of pathways with the potential targets to evaluate the key targets [37].

### 2.7. Compound–Target–Signaling Pathway Network

The compound–target–signaling pathways network was built by integration of compound–target and pathway–target networks. The Cytoscape program was used to understand the interaction of active constituents with prospective targets and signaling pathways in order to evaluate the primary targets.

### 2.8. Molecular Docking

Molecular docking makes it easier to figure out how ligands interact with their corresponding proteins. Finally, the results of this network pharmacological study were verified through molecular docking approach. For that, the 3D structures of active components were retrieved from the PubChem search in the .sdf format and optimized [43]. The Research Collaboratory for Structural Bioinformatics Protein Data Bank (RCSBPDB) (https://www.rcsb.org/, accessed on 29 August 2022) was used to retrieve receptor protein data, and their PDB files were downloaded [44]. The input protein was preprocessed by the Chimera to eliminate ligand molecules from the source file in order to fulfill the docking criteria.

**Figure 1:**
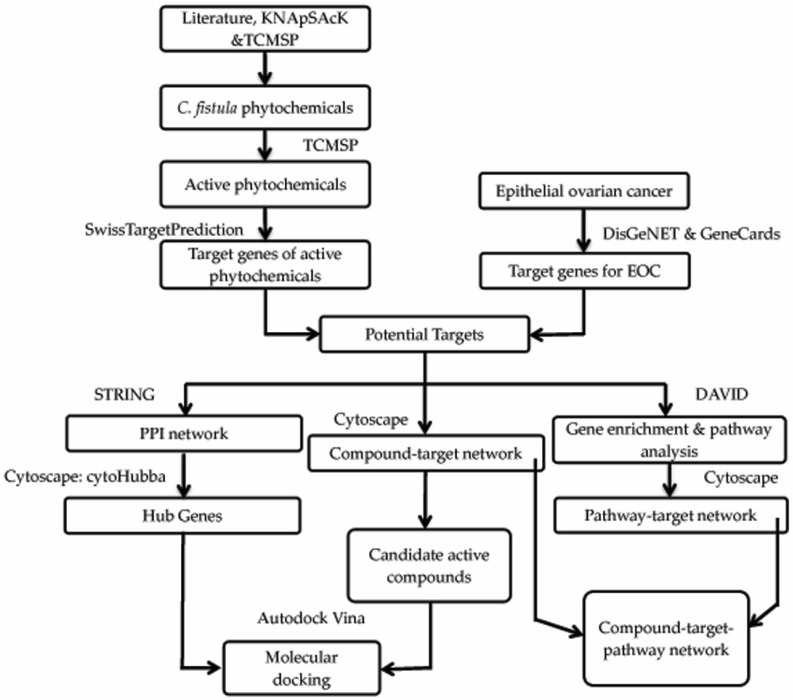
The network pharmacology and molecular docking techniques employed in order to predict *C. fistula’s* potential drug targets for the treatment of EOC depicted graphically.

Auto-dock Vina is used to carry out the protein–ligand docking for the prediction of predominant binding mode of the ligand with a protein [45]. The active constituents acted as ligands, while the targets acted as receptors. Finally, the best docking results were selected for the visualization using the Chimera. The graphical framework of the network pharmacology and molecular docking techniques is shown (Figure 1).

## 3. Results

### 3.1. Active Constituents of C. fistula

A phytochemical library was constructed with the help of previous knowledge and multiple databases. That library contained 78 phytochemicals isolated from different parts of *C. fistula* such as leaves, fruit, bark, stem, as well as seeds (Table S1). Eight phytochemicals (Rhein, Ellagic Acid, Quercetin, Kaempferol, Gibberellin A3, Licoisoflavone, β-Sitosterol and Stigmasterol) were predicted with pharmacokinetic criteria (≥30% OB and ≥0.18 DL. These eight phytochemicals were filtered as effective constituents and their properties are presented (Table 1).

### 3.2. Drug Target Profiling for C. fistula

In total, 800 target genes of corresponding active phyto-constituents of *C. fistula* were collected from the SwissTargetPrediction that showed the bioactivity of active constituents of *C. fistula*. Protein ID’s of the targets were aligned from UniProtKB to remove the duplicates. In the end, 415 unique targets are selected for further analysis.

### 3.3. Potential Targets of C. fistula for EOC

**Figure 2:**
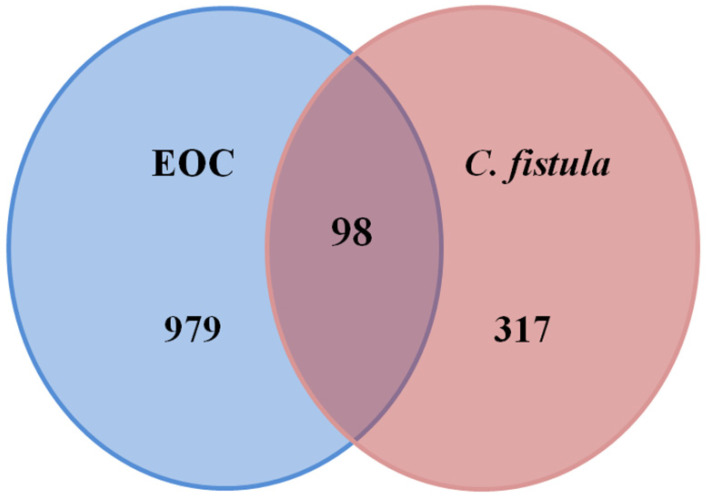
Venn diagram of potential targets.

A total of 1077 EOC-related targets (Table S2) were acquired by using DisGeNET & Genecard databases. These were intersected to the active constituent’s target genes. Out of those, 98 potential targets (Table S3) were predicted for the treatment of EOC (Figure 2).

### 3.4. Compound–Target Network

**Figure 3:**
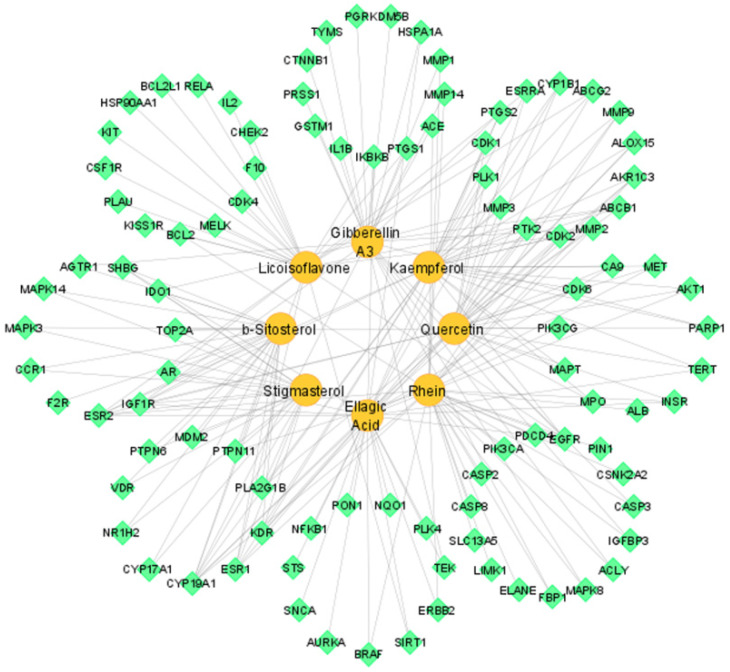
Compound–target network of active constituents and potential targets (circle shape shows active constituents and diamond shape shows potential targets).

The compound–target network of eight active constituents to corresponding 98 potential targets was constructed by using Cytoscape software. That network had 106 nodes and 217 edges. Each node represented active constituents or potential targets, and lines showed interaction between them (Figure 3).

Topological analysis of network revealed the network characteristics: the density is 0.039, network centralization is 0.271, network heterogeneity is 1.668, and characteristic path length is 3.042. Furthermore, the active constituents with respective degrees were found as: Rhein (32), Ellagic Acid (32), Quercetin (30), Kaempferol (29), Gibberellin A3 (28), Licoisoflavone (26), β-Sitosterol (21), and Stigmasterol (19). They interacted with multiple targets (Table 2).

### 3.5. Protein–Protein Interaction (PPI) Network

**Figure 4:**
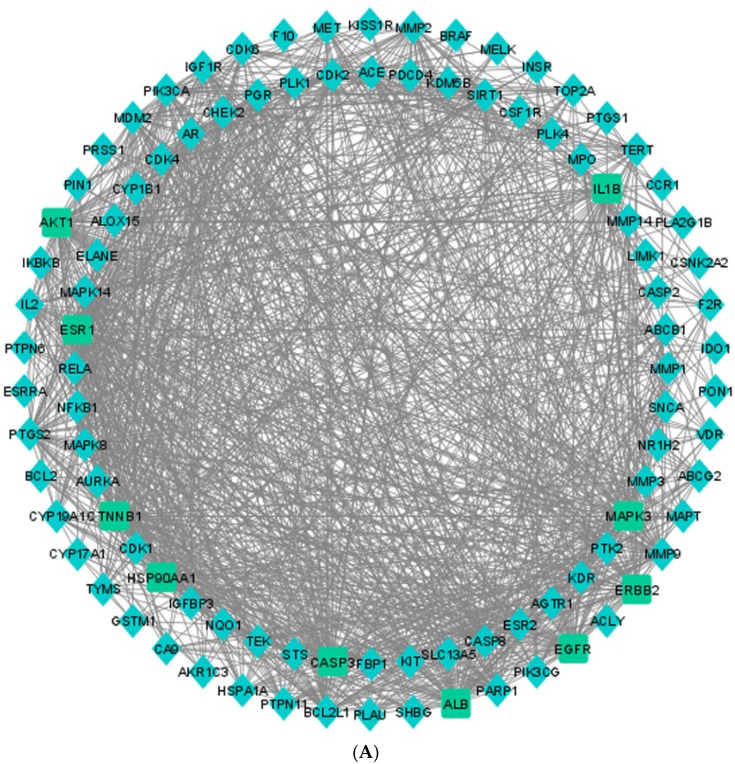

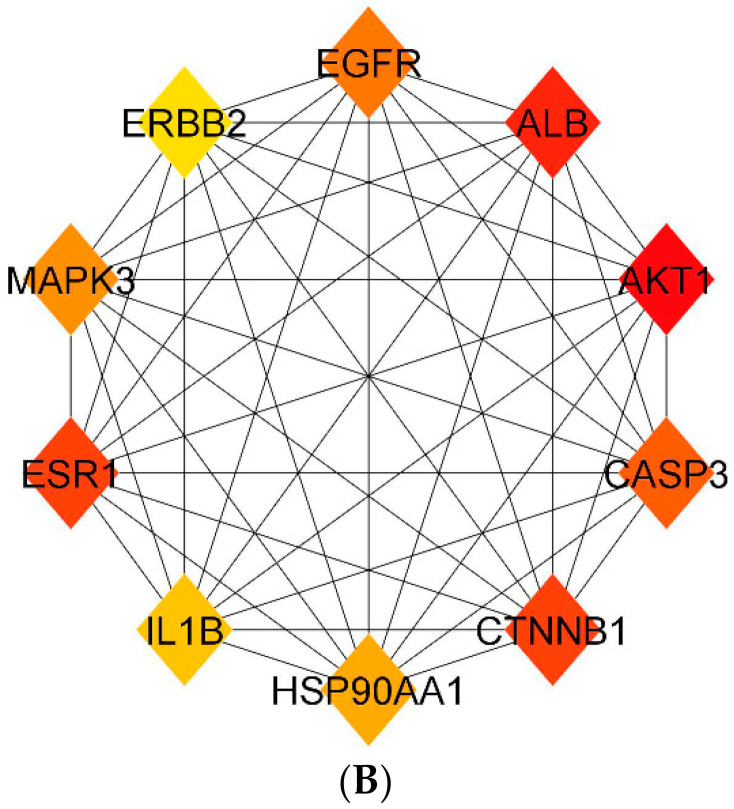
(**A**) Analysis and Visualization of PPI network in Cytoscape. (**B**) Top 10 targets of *C. fistula* on EOC analyzed by Cytoscape.

The 98 potential target genes of *C. fistula* which may be the potential targeting genes to treat EOC were copied to STRING v11.5to build a PPI network with a score of 0.4 confidence interaction. That was performed to predict the interactions of potential proteins with *Homo sapiens* proteins and their physiological functions. In that network, the nodes are representing the targets, and the lines connecting the nodes are edges that represent the intermolecular interactions between multiple targets during a disease development (Figure S1). There were 98 nodes and 1153 edges in the network. In addition, the density, heterogeneity, network centralization, and path length were 0.243, 0.721, 0.521, and 1.862, respectively (Figure 4A). Later, the “Network Analyzer” tool was used to examine the PPI network. The five highest degree targets were AKT1 (73), ALB (65), CTNNB1 (64), ESR1 (64), and CASP3 (62). The higher degree depicted that the target genes are extremely connected; hence, these five genes might be important targets (Figure 4B). When the data were compared to the data obtained from enrichment analysis of 98 potential targets, specifically four out of those five genes, AKT1, CTNNB1, ESR1, and CASP3, were identified as the principal anticancerous targets of *C. fistula.*

### 3.6. Gene Functional Annotation

**Figure 5:**
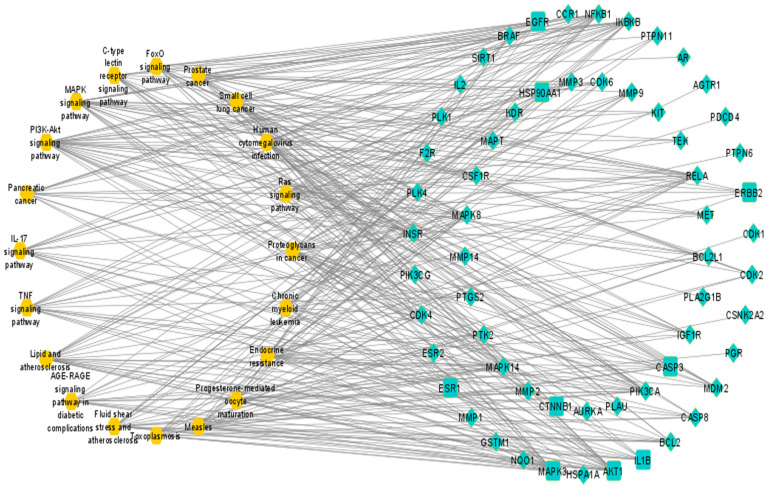
Hub genes enriched in top 20 signaling pathways. Signaling pathways are represented as hexagons and targets are represented as ellipses.

**Figure 6:**
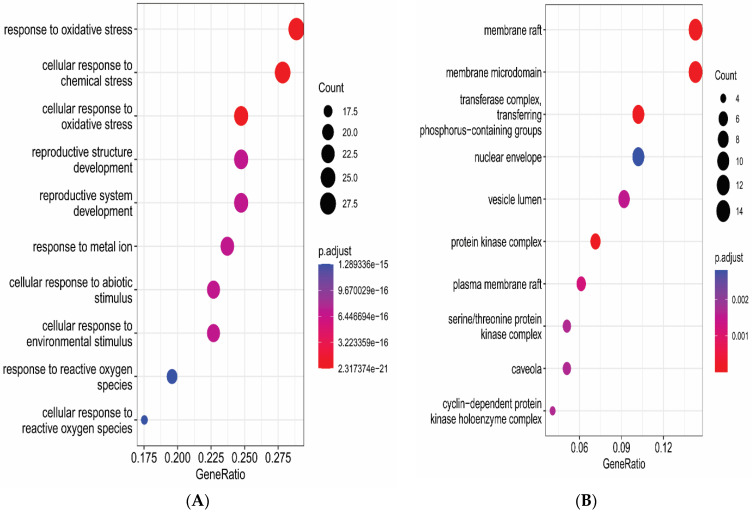

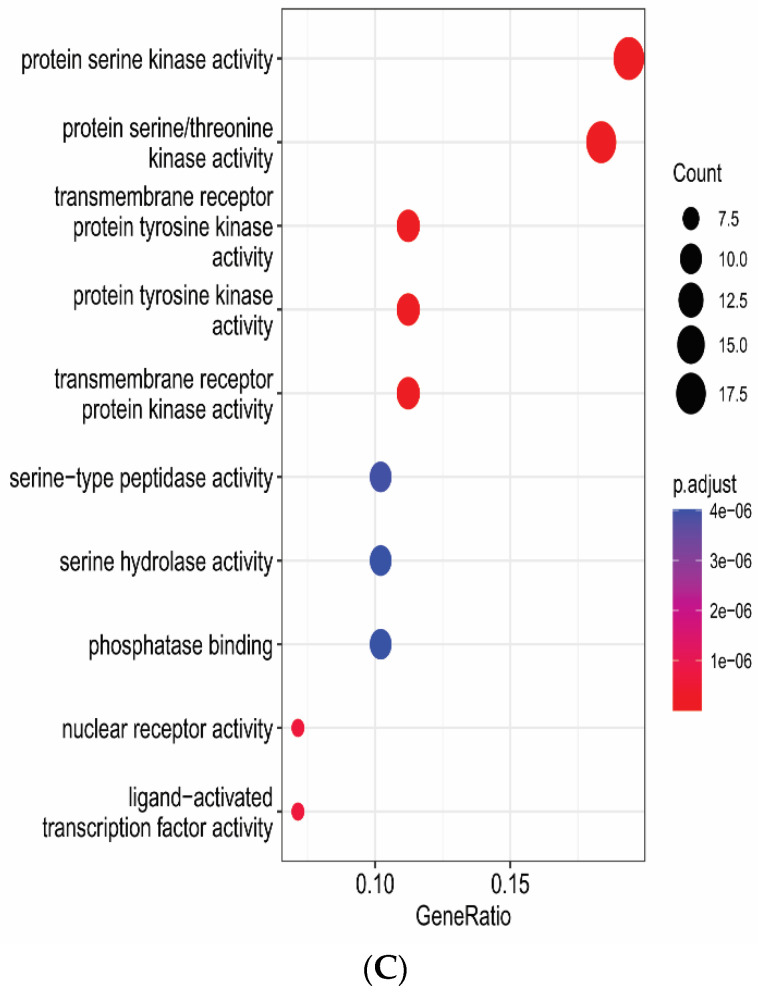
GO analysis of *C. fistula*’s potential targets on EOC. (**A**) Biological Processes (BP), (**B**) Cellular Components (CC), and (**C**) Molecular Functions (MF).

**Figure 7:**
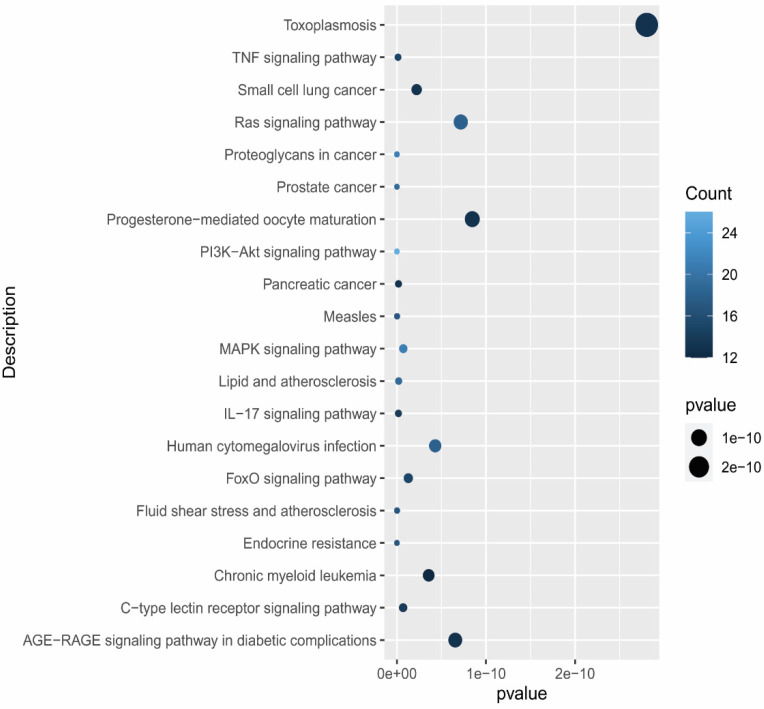
KEGG pathway enrichment analysis: 20 enriched pathways plotted through R language.

The gene and enriched pathways were analyzed through DAVID to predict gene role and signaling pathways of eight active constituents of *C. fistula* for the EOC treatment. The GO enrichment analysis contained 324 Biological Processes (BPs), 51 Cellular Components (CCs) and 85 Molecular Functions (MFs) as conformed screening criteria, with count ≥ 2 and *p* ≤ 0.05. Moreover, 133 enriched pathways were identified on the basis of criteria *p* < 0.05. A pathway–target network was constructed by using Cytoscape (Figure 5). GO annotations and KEGG pathway were plotted by using ggplot2 in R language (Figure 6 and Figure 7).

### 3.7. Compound–Target–Signaling Pathway Network

**Figure 8:**
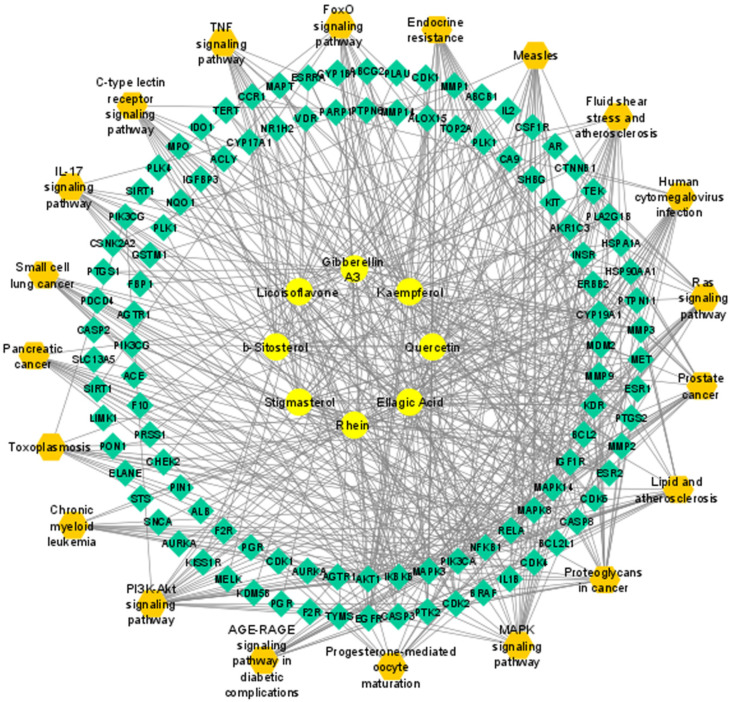
*C. fistula*’s compound–target–signaling pathway network to EOC. Candidate active phytochemicals shown as ellipses, potential targets represented as diamonds, and pathways represented as hexagons.

A compound–target–signaling pathways network was built by integration of compound–target and pathway–target networks using the Cytoscape. The “Network Analyzer” analysis showed that it included 126 nodes, 570 edges, 8 active phytochemicals, 98 potential targets and 20 associated pathways. The targets of active phytochemicals were interconnected with pathways (Figure 8).

### 3.8. Molecular Docking

**Figure 9:**
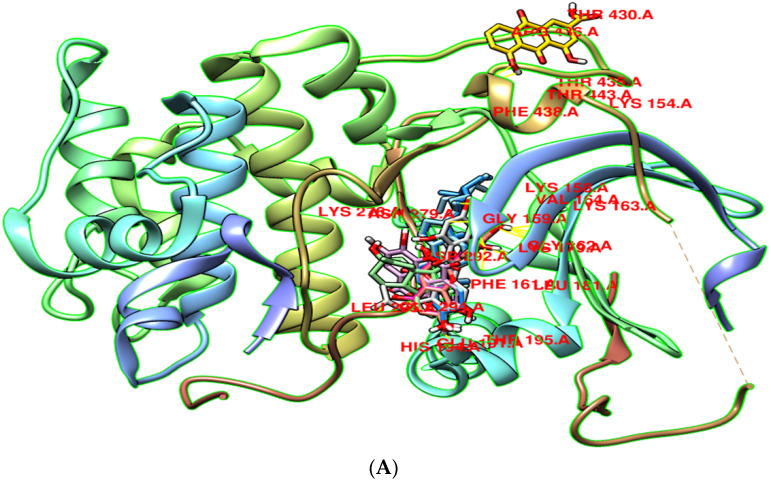

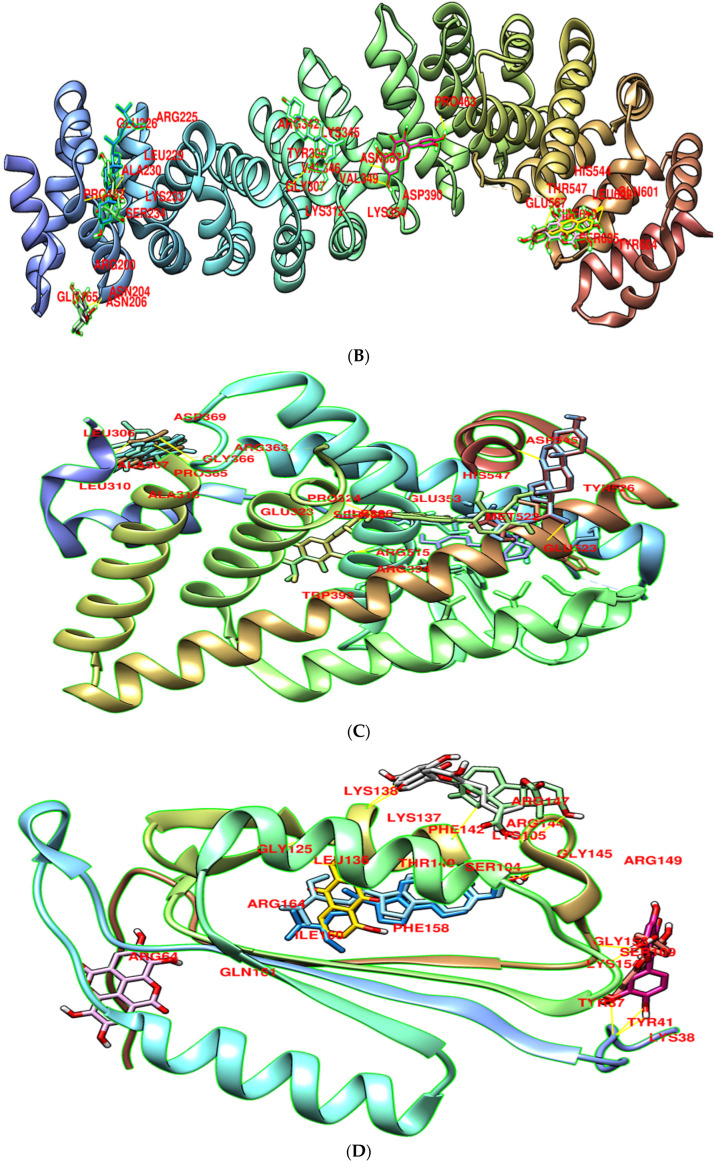
The binding site residues with the four proteins are shown in the docking complex of four targets with their best binding components: (**A**) AKT1; (**B**) CTNNB1; (**C**) ESR1; (**D**) CASP3.

Molecular docking was used to screen potential targets of components with the capacity to decrease the prevalence of EOC. The highest four target genes, AKT1, CTNNB1, ESR1, and CASP3 were selected via the topological examination of PPI network. The three-dimensional (3D) structures of these target proteins (AKT1 (PDB id: 3QKK), CTNNB1 (PDB id: 1jdh), ESR1 (PDB id: 1pcg), CASP3 (PDB id: 3kjf) were retrieved through the PDB. These structures were refined by the UCSF Chimera tool with 1000 decent steps of energy minimization [46]. In order to avoid collisions and erroneous compositions, non-standard residues were eliminated from the proteins. The docking analysis was used to accurately predict the significant binding affinity between active constituents and four target protein’s’ binding pockets. The eight active components of *C. fistula* were docked with the four EOC potential targets (Figure 9). All compounds and targets showed binding scores ranging from −5.8 to −9.2 kcal/mole. According to the docking studies, eight active constituents reported greater binding energy with key EOC targets. Among those, more specifically, two phytochemicals, β- Sitosterol and Stigmasterol, were prominent (Table 3).

## 4. Discussion

The etiology and pathogenesis of EOC is very complex and not yet fully understood. It is characterized by sneaking symptoms. That is why individuals are examined at a later stage. Cyto-reduction surgeries (CRS) are used to treat this, followed by platinum-based chemotherapy [1]. These types of medications incorporate adverse side effects on the individual’s well-being [2]. Owing to the progress in the treatment of OC, targeted medicines are available now. Nevertheless, targeted/focused medications are expensive and the options are frequently restricted. Therefore, more effective, safe, and affordable therapeutic medications are urgently needed for the OC.

Natural phytochemicals, especially plant-derived compounds, are widely employed as alternative therapeutics, such as taxol for cancers [47]. The reason is that they are inexpensive, more accessible, multi-targeting, and contain low toxicity. Herbal medicines have been utilized in order to cure human illness since ancient times and are thought to be the rich origin of drug discovery. *C. fistula* is a versatile plant that has shown the antioxidant, anti-inflammatory, immunological, hepatoprotective, antipyretic, analgesic, and antitumor properties [30]. More than 60 phytochemicals, including flavonoids, anthraquinones, chromones, coumarins, alkaloids, phytosterols, long-chain hydrocarbons, phenolic, and other phytochemicals have been found in *C. fistula.*

This study has provided a baseline for the screening of *C. fistula*’s bioactive compounds. That was a unique therapeutic idea for future research of *C. fistula*’s processes for EOC treatment. Anthraquinone, polyphenols, hormones, phytosterols, and flavonoids were the most abundant bioactive chemicals discovered in *C. fistula*. These played a critical role in the development of EOC. This is a strong indicator that a number of targets may work together to provide a synergistic impact.

By PPI network topological analysis, the four key targets AKT1, CTNNB1, ESR1, and CASP3 were evaluated for anti-EOC activity. This is a strong indicator that a number of targets may work together to provide a synergistic impact. AKT1 promotes OC cell proliferation, immigration, epithelial transformation to mesenchyme (EMT), gluconeogenesis, and resistance to therapeutics and serves as a biomarker for OC therapy response [48,49,50,51]. CTNNB1 is involved in the growth of OC due to its deregulated impact [52,53,54]. ESR1 is implicated in amplification and penetration, and its hereditary characteristics are linked to OC probability and advancement [55,56,57]. CASP3 activity and expression are linked to cell death, immigration, amplification, susceptibility of OC cells to anticarcinogenic drugs, as well as metastasis and prognostic outcomes in OC patients [55,56,57,58].

Gene enrichment analysis reported that anti-EOC target genes of *C. fistula* were primarily involved in different molecular processes such as protein serine activity, protein serine/threonine kinase activity, transmembrane receptor protein tyrosine kinase activity, and transmembrane receptor protein kinase activity. The pathway enrichment analysis evaluate that genes were focused to EOC-associated signaling pathways: the ‘PI3K-Akt signaling pathway, proteoglycans in cancer, the TNF signaling pathway, and the IL-17 signaling pathway. The PI3K-Akt signaling pathway might boost tumor progression, amplification, and uncontrolled mitosis OC cells [56,57,59]. Proteoglycans in cancer modulate adhesion and migration are also associated to mutagenic and angiogenic growth factors [60]. TNF signaling pathways promote unregulated inflammation, which can be suppressed with medication, and their expression profiles are linked to OC cancer [61,62]. The pro-inflammatory cytokine interleukin-17 (IL-17) has been linked to low tumor grade [63].

Through the network analysis, the four main targets AKT1, CTNNB1, ESR1, and CASP3 were evaluated for their binding energies to eight active components of *C. fistula*. It exemplifies the expression of multiple targets, components, and pathways. In molecular docking, more negative the binding energy, the greater the expected affinity for binding of the ligand to the target [64]. The β-Sitosterol showed lower binding affinities to AKT1 and CTNNB1. Stigmasterol interacted to AKT1 and ESR1 with lower binding affinities. This revealed that *C. fistula* has anti-EOC properties, inhibiting EOC main targeting genes. Network pharmacology integrated to molecular docking approach appeared to be effective for the identification of biologically active phytochemicals, associated potential genes, and linked signaling pathways to treat EOC. It provided a systematic framework for subsequent investigation of phytochemicals against a wide range of disorders.

## 5. Conclusions

This research set the foundation to establish the efficacy of multicomponent, multitarget chemical formulae and identification of genes that target to treat EOC. Network pharmacology and molecular docking approaches were incorporated in order to reveal the underlying mechanisms of *C. fistula* to treat EOC. Furthermore, our data suggest that the AKT1, CTNNB1, ESR1, and CASP3 targets are intriguingly beneficial to slow down the prevalence of EOC, potentially resulting in therapeutic benefits in EOC. This is the first time four genes have been reported against the EOC from *C. fistula*. However, more pharmacological and clinical studies are required to confirm our findings. This method lays the framework for future research into *C. fistula* for EOC-protective mechanisms and the use of network pharmacology in drug development.

## Figures and Tables

**Table 1:** Properties of active phytochemicals *C. fistula*.

Sr.No.	Phytochemicals	Molecular Formula	Molecular Weight (Dalton)	Drug Likeness (>0.18)	Oral Bioavailability (>30%)	2D Structures	PubChem ID
01	Rhein	C_15_H_8_O_6_	284.22	0.28	47.07	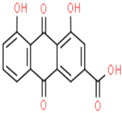	10168
02	Ellagic Acid	C_14_H_6_O_8_	302.19	0.43	43.06	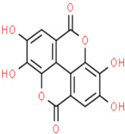	5281855
03	Quercetin	C_15_H_10_O_7_	302.23	0.28	46.43	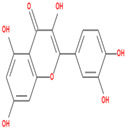	5280343
04	Kaempferol	C_15_H_10_O_6_	286.24	0.24	41.88	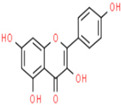	5280863
05	Gibberellin A3	C_19_H_22_O_6_	346.4	0.53	81.59	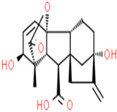	6466
06	Licoisoflavone	C_20_H_18_O_6_	354.4	0.42	41.61	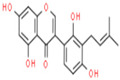	5281789
07	β-Sitosterol	C_29_H_50_O	414.7	0.75	36.91	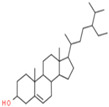	222284
08	Stigmasterol	C_29_H_48_O	412.7	0.76	43.83	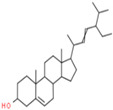	5280794

**Table 2:** Degrees of 8 active constituents analyzed by the Cytoscape.

Sr. No.	Phytochemical Name	Categories	Degree
01	Rhein	Anthraquinone	32
02	Ellagic Acid	Polyphenol	32
03	Quercetin	Flavonoid	30
04	Kaempferol	Flavonoid	29
05	Gibberellin A3	Hormone	28
06	Licoisoflavone	Flavonoid	26
07	ß-Sitosterol	Phytosterols	21
08	Stigmasterol	Phytosterols	19

**Table 3:** The binding affinities of prospective target genes with phytochemicals in the docking analysis.

Sr. No	Compound	Binding Affinities (kcal/mol)			
		AKT1	CTNNB1	ESR1	CASP3
01	Rhein	−7.5	−6.9	−6.8	−6.2
02	Ellagic acid	−7.9	−7.2	−6.6	−6.4
03	Quercetin	−7.7	−6.5	−7.7	−6.1
04	Kaempferol	−7.6	−6.7	−7.3	−5.8
05	Gibberelin A3	−8.2	−6.7	−6.6	−6.7
06	Licoisoflavone	−7.9	−6.1	−6.6	−6.5
07	β- Sitosterol	−8.8	−7.0	−6.7	−6.8
08	Stigmasterol	−9.2	−6.7	−7.0	−6.8

## Data Availability

Not applicable.

## Supplementary Materials

The following supporting information can be downloaded at: https://www.mdpi.com/article/10.3390/pharmaceutics14091970/s1. Figure S1: PPI network constructed by STRING. It represents the interaction of potential genes of *C. fistula* against EOC. Table S1: Phytochemical library of *C. fistula*. Table S2: The unique genes and variants of EOC fetched from DisGeNET & Genecards databases. Table S3: Potential targets of *C. fistula* for EOC.
