# Supplementary material for: Molecular Mechanisms of Cassia fistula against Epithelial Ovarian Cancer Using Network Pharmacology and Molecular Docking Approaches
Source: Pharmaceutics. 2022 Sep 19;14(9):1970. doi: 10.3390/pharmaceutics14091970 (PMC9500712; doi:10.3390/pharmaceutics14091970)
Supplement: Supplementary file 1 [file pharmaceutics-14-01970-s001.zip › pharmaceutics-1865785-supplementary.pdf]

Supplementary Materials: Molecular Mechanisms of *Cassia fistula* against Epithelial Ovarian Cancer Using Network Pharmacology and Molecular Docking Approaches

Aqsa Kanwal [1,†], Farrukh Azeem [1,†], Habibullah Nadeem [1], Usman Ali Ashfaq [1], Rana Muhammad Aadil [2], A. K. M. Humayun Kober [3], Muhammad Shahid Riaz Rajoka [4], Ijaz Rasul [1,*]

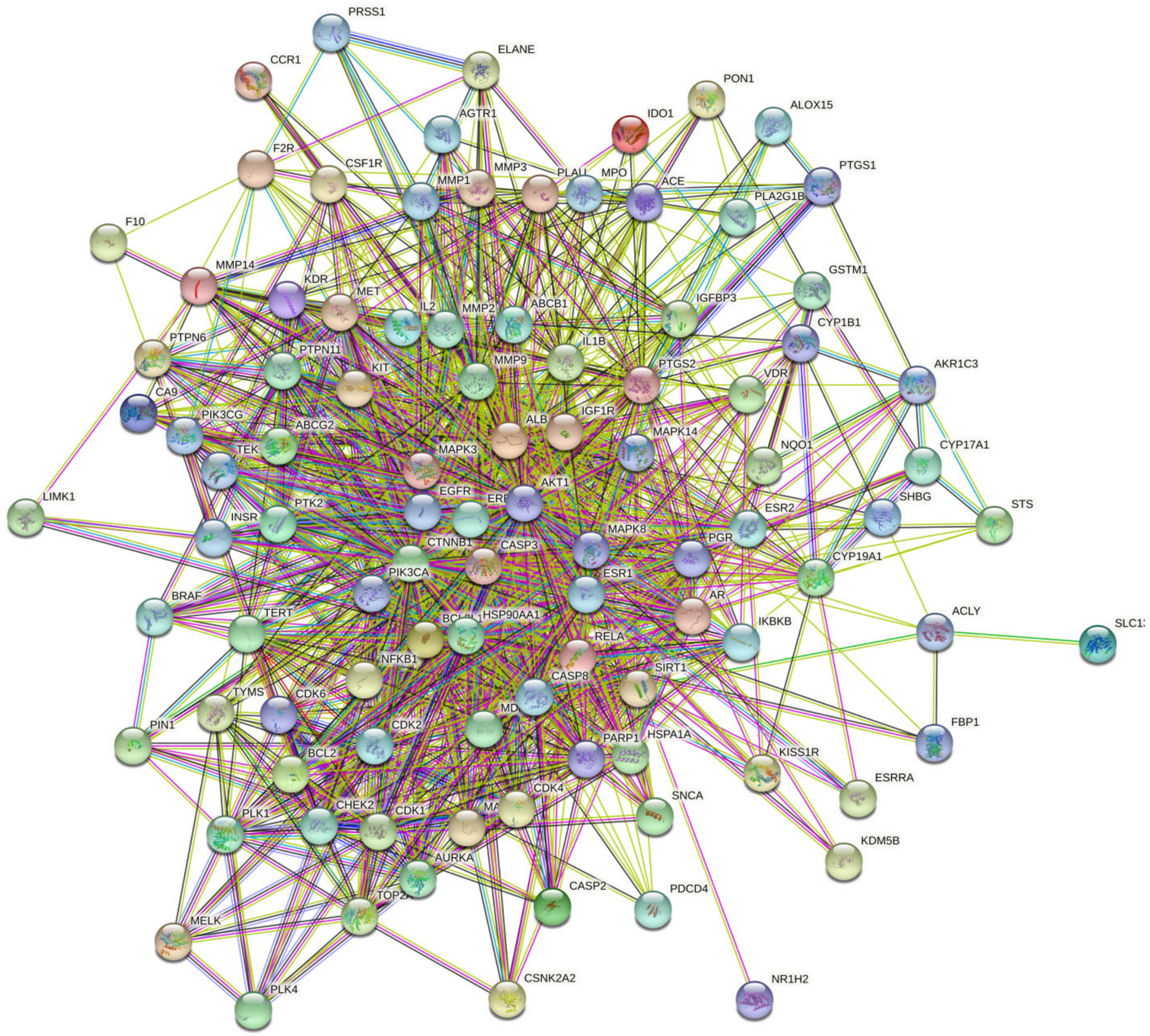

Figure S1. PPI network constructed by STRING. It represents the interaction of potential genes of *C. fistula* against EOC.

Table S1. Phytochemical library of *C. fistula.*

Sr. No.	Phytochemical Names	Mol. Formula	Mol. Weight (Dalton)	OB	DL	PubChem ID	2D structure
01	3,4-di-O-caffeoylquinic acid	$C_{25}H_{24}O_{12}$	516.46	1.78	0.69	5281780	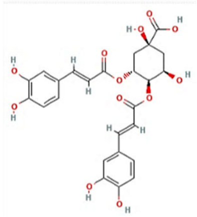
02	HOP-22(29)-EN-3. BETA.-OL	$C_{30}H_{50}O$	426.7	-	-	604951	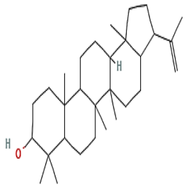
03	2,5-Dimethyl-7-hydroxychromone	$C_{11}H_{10}O_3$	190.2	16.13	0.08	5316891	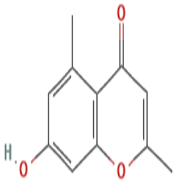
04	apigenin-6,8-di-C-glycoside	$C_{27}H_{30}O_{15}$	594.52	3.42	0.78	442666	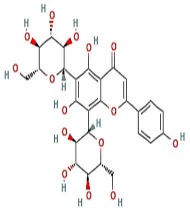
05	1-Octacosanol	$C_{28}H_{58}O$	410.8	10.70	0.41	68406	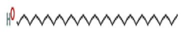

06	5 hydroxymethylfurfural	$C_6H_6O_3$	126.11	45.07	0.02	237332	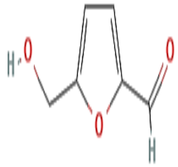
07	Tetratetracontane	$C_{44}H_{90}$		7.82	0.25	23494	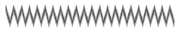
08	Pentadecanal	$C_{15}H_{30}O$	226.40	10.38	0.06	17697	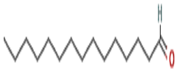
09	Phytol	$C_{20}H_{40}O$		33.82	0.13	5280435	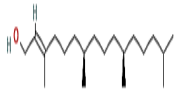
10	Octacosane	$C_{28}H_{58}$	394.77	8.15	0.37	12408	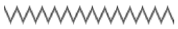
11	7-hexadecyne	$C_{16}H_{30}$	222.42	4.21	0.06	549042	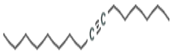

12	Lupeol	$C_{30}H_{50}O$	426.73	12.12	0.78	259648	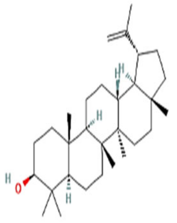
13	Dichloromethane	CH_2Cl_2	84.93	7.57	0.00	6344	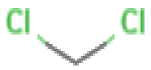
14	Kaempferol	$C_{15}H_{10}O_6$	286.24	41.88	0.24	5280863	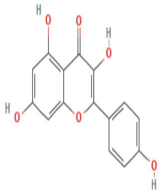
15	Procyanidin B2	$C_{30}H_{26}O_{12}$	578.53	3.01	0.66	122738	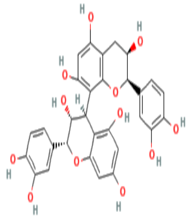
16	Biochanin A	$C_{16}H_{12}O_5$	284.27	25.21	0.24	5280373	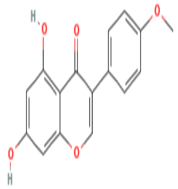
17	Licoisoflavone	$C_{20}H_{18}O_6$	354.36	41.61	0.42	5281789	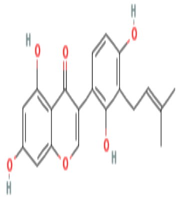

18	Amentoflavone	$C_{30}H_{18}O_{10}$	538.46	2.95	0.65	5281600	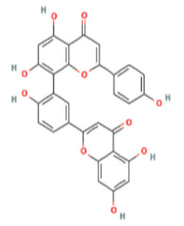
19	Chrysophanol	$C_{15}H_{10}O_4$	254.24	18.64	0.21	10208	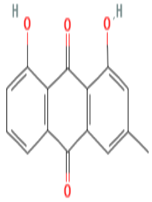
20	Rhein	$C_{15}H_8O_6$	284.22	47.07	0.28	10168	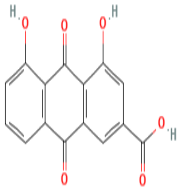
21	Ellagic Acid	$C_{14}H_6O_8$	302.19	43.06	0.43	5281855	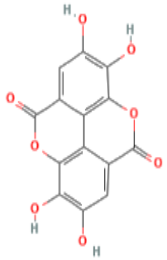
22	Quercetin	$C_{15}H_{10}O_7$	302.23	46.43	0.28	5280343	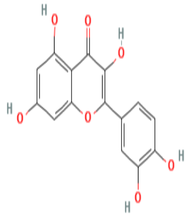
23	Gibberellin A3	$C_{19}H_{22}O_6$	346.4	81.59	0.53	6466	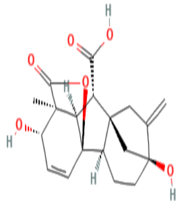

24	ß-Sitosterol	$C_{29}H_{50}O$	414.7	36.91	0.75	222284	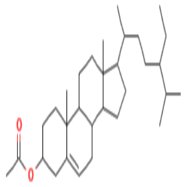
25	Stigmasterol	$C_{29}H_{48}O$	412.7	43.83	0.76	5280794	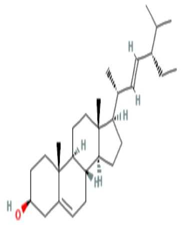
26	Emodin	$C_{15}H_{10}O_5$	270.24	24.40	0.24	3220	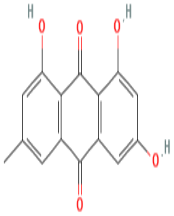
27	Physcion	$C_{16}H_{12}O_5$	284.27	22.29	0.27	10639	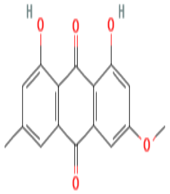
28	Citreorosein	$C_{15}H_{10}O_6$	286.24	22.19	0.27	361512	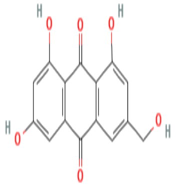
29	Ziganein	$C_{15}H_{10}O_4$	254.24	18.92	0.21	5316800	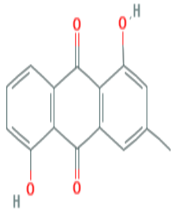

30	Sennoside A	$C_{42}H_{38}O_{20}$	862.75	3.34	0.08	73111	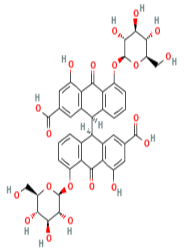
31	Sennoside B	$C_{42}H_{38}O_{20}$	862.75	3.34	0.08	91440	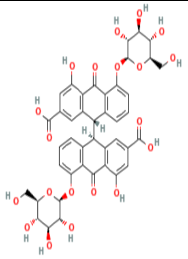
32	Isoscopoletin	$C_{10}H_8O_4$	192.17	23.46	0.08	69894	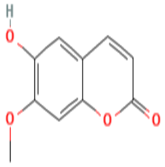
33	Scopoletin	$C_{10}H_8O_4$	192.17	27.77	0.08	5280460	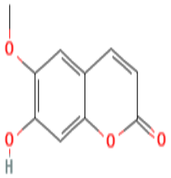
34	Esculetin	$C_9H_6O_4$	178.14	22.97	0.07	5281416	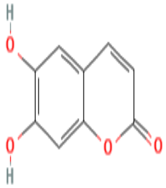
35	Cherianoine	$C_{12}H_{13}NO_4$	235.24	27.32	0.12	5315819	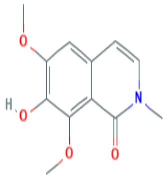

36	Isovanillic acid	$C_8H_8O_4$	168.15	39.42	0.04	12575	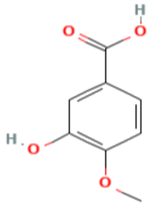
37	Vanillic acid	$C_8H_8O_4$	168.15	35.47	0.04	8468	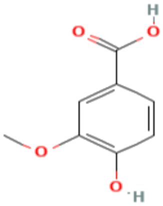
38	Clerosterol	$C_{29}H_{48}O$	412.7	7.56	0.76	5283638	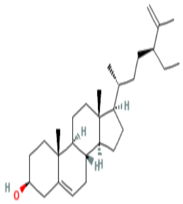
39	Stearic acid	$C_{18}H_{36}O_2$	284.48	17.83	0.14	5281	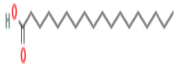
40	Oleic acid	$C_{18}H_{34}$O2	282.47	33.13	0.14	445639	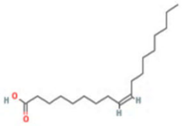
41	Linoleic acid	$C_{18}H_{32}O_2$	280.45	41.90	0.14	5280450	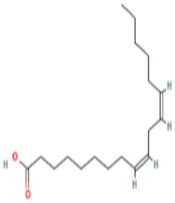

42	Lutein	$C_{40}H_{56}O_2$	568.89	22.59	0.55	5281243	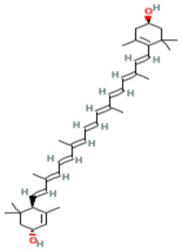
43	Pyruvic acid	$C_3H_4O_3$	88.06	54.46	0.01	1060	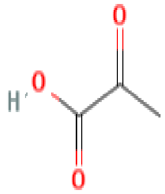
44	Valeric acid	$C_5H_{10}O_2$	102.13	70.74	0.01	7991	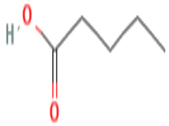
45	Scopolin	$C_{16}H_{18}O_9$	354.31	25.50	0.39	439514	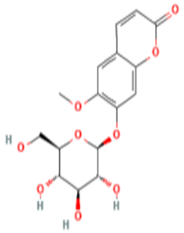
46	Uridine	$C_9H_{12}N_2O_6$	244.2	10.49	0.11	6029	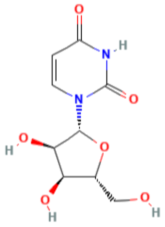
47	1H-indole-3-ethanamine	$C_{10}H_{12}N_2$	160.22	32.08	0.05	1150	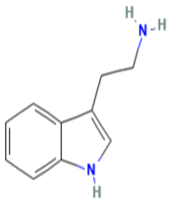

48	L-Norvaline	$C_5H_{11}NO_2$	117.15	75.82	0.01	65098	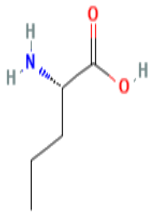
49	Benzenepropanoic acid	$C_9H_{10}O_2$	150.18	35.83	0.03	107	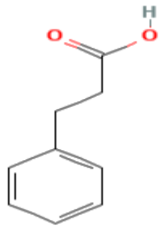
50	n-Cetane	$C_{16}H_{34}$	226.45	12.32	0.06	11006	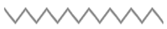
51	Octadecane	$C_{18}H_{38}$	254.5	9.81	0.09	11635	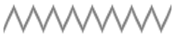
52	Cumin aldehyde	$C_{10}H_{12}O$	148.2	38.29	0.03	326	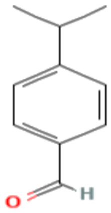
53	Aromadendrin	$C_{15}H_{12}O_6$	288.25	23.04	0.24	122850	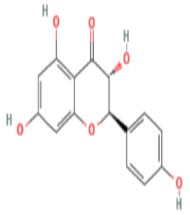

54	Epicatechin	$C_{15}H_{14}O_6$	290.27	28.93	0.24	72276	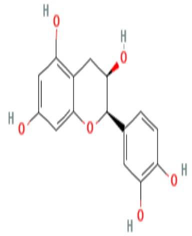
55	Caffeic Acid	$C_9H_8O_4$	180.16	25.76	0.05	689043	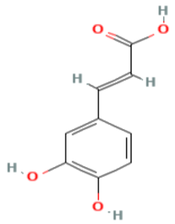
56	Rutin	$C_{27}H_{30}O_{16}$	610.52	3.20	0.68	5280805	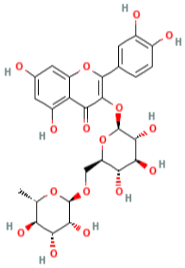
57	Eriodictyol-7-O-neohesperidoside	$C_{27}H_{32}O_{15}$	596.54	3.93	0.73	114627	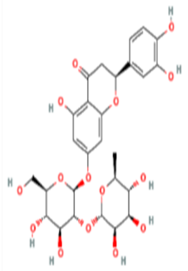
58	Triacontane	$C_{33}H_{68}$	422.83	8.09	0.41	12535	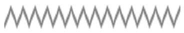
59	Chrysophanein	$C_{21}H_{20}O_9$	416.38	20.06	0.76	6324923	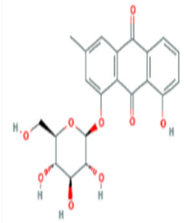

60	16-Heptadecenal	$C_{17}H_{32}O$	252.44	28.87	0.10	557525	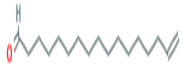
61	Ceryl Alcohol	$C_{26}H_{54}O$	382.72	10.95	0.35	68171	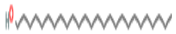
62	Sweroside	$C_{16}H_{22}O_9$	358.34	4.96	0.38	161036	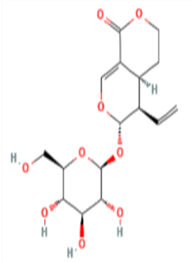
63	Umbelliferone	$C_9H_6O_3$	162.14	27.37	0.05	5281426	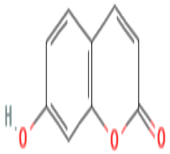
64	Nonacosane	$C_{29}H_{60}$	408.8	8.12	0.39	12409	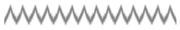
65	Rubiadin	$C_{15}H_{10}O_4$	254.24	25.02	0.21	124062	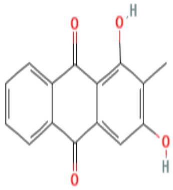

66	Heptacosanoic Acid	$C_{27}H_{54}O_2$	410.73	13.96	0.44	23524	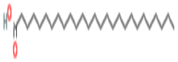
67	Malvalic acid	$C_{18}H_{32}O_2$	280.45	30.99	0.15	10416	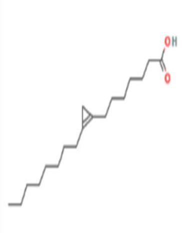
68	Galactomannan	$C_{18}H_{32}O_{16}$	504.44	10.92	0.70	439336	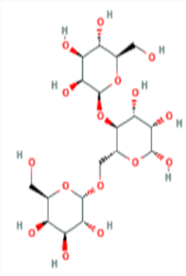
69	Oxalic Acid	$C_2H_2O_4$	90.03	29.68	0.01	971	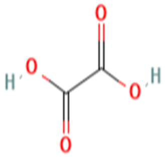
70	Sterculic acid	$C_{19}H_{34}O_2$	294.48	29.77	0.18	12921	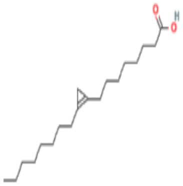
71	Myristic acid	$C_{14}H_{28}O_2$	228.38	21.18	0.07	11005	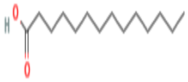

72	Gallic Acid	$C_7H_6O_5$	170.12	31.69	0.04	370	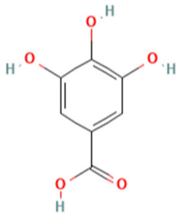
73	Pectin	$C_6H_{10}O_7$	194.14	40.39	0.06	441476	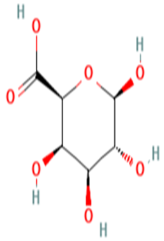
74	Dihydrokaempferol	$C_{15}H_{12}O_6$	288.25	23.04	0.24	122850	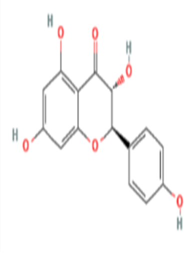
75	Butyric Acid	$C_4H_8O_2$	88.11	21.62	0.00	264	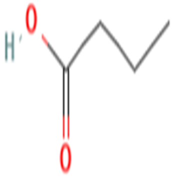
76	Caprylic acid	$C_8H_{16}O_2$	144.21	16.40	0.02	379	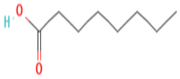
77	Formic acid	CH_2O_2	46.03	33.26	0.00	284	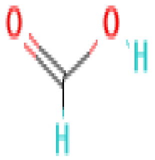

78	Aloin	$C_{21}H_{22}O_9$	418.4	11.80	0.71	12305761	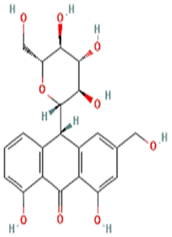

Table S2. The unique genes and variants of EOC fetched from DisGeNET & Genecards databases.

Sr. No.	Gene	Gene name	Protein class	Gene ID
1	MMP2	matrix metallopeptidase 2	Enzyme	4313
2	EDN1	endothelin 1	Signaling	1906
3	CXCR4	C-X-C motif chemokine receptor 4	G-protein coupled receptor	7852
4	MIR20A	microRNA 20a		406982
5	MIRLET7G	microRNA let-7g		406890
6	MMP7	matrix metallopeptidase 7	Enzyme	4316
7	ZEB1	zinc finger E-box binding homeobox 1	Transcription factor	6935
8	CDK14	cyclin dependent kinase 14	Kinase	5218
9	SOD2	superoxide dismutase 2	Enzyme	6648
10	SEMA4D	semaphorin 4D	Signaling	10507
11	MIR200B	microRNA 200b		406984
12	BRCA1	BRCA1 DNA repair associated	Enzyme	672
13	LINC00460	long intergenic non-protein coding RNA 460		728192
14	TEK	TEK receptor tyrosine kinase	Kinase	7010
15	MIR630	microRNA 630		693215
16	PDCD4	programmed cell death 4	Nucleic acid binding	27250
17	FOXP3	forkhead box P3		50943
18	SMYD3	SET and MYND domain containing 3	Epigenetic regulator	64754
19	ROR2	receptor tyrosine kinase like orphan receptor 2	Kinase	4920
20	FOXM1	forkhead box M1		2305
21	CDK1	cyclin dependent kinase 1	Kinase	983
22	PLXNA1	plexin A1		5361
23	CD82	CD82 molecule		3732
24	MIR193A	microRNA 193a		406968
25	ID1	inhibitor of DNA binding 1, HLH protein	Transcription factor	3397
26	PIK3CB	phosphatidylinositol-4,5-bisphosphate 3-kinase catalytic subunit beta	Kinase	5291
27	COL18A1	collagen type XVIII alpha 1 chain		80781
28	ASS1	argininosuccinate synthase 1	Enzyme	445
29	RGS4	regulator of G protein signaling 4	Enzyme modulator	5999
30	ADAM23	ADAM metallopeptidase domain 23	Enzyme	8745
31	CYP1A1	cytochrome P450 family 1 subfamily A member 1	Enzyme	1543
32	KDM4B	lysine demethylase 4B	Epigenetic regulator	23030

33	GSTP1	glutathione S-transferase pi 1		2950
34	MIR122	microRNA 122		406906
35	ADRA1A	adrenoceptor alpha 1A	G-protein coupled receptor	148
36	MIR206	microRNA 206		406989
37	BST1	bone marrow stromal cell antigen 1	Enzyme	683
38	KISS1R	KISS1 receptor	G-protein coupled receptor	84634
39	IL18BP	interleukin 18 binding protein		10068
40	MAD2L1	mitotic arrest deficient 2 like 1		4085
41	DLEC1	DLEC1 cilia and flagella associated protein		9940
42	HP	haptoglobin	Enzyme	3240
43	MIR628	microRNA 628		693213
44	PPARGC1A	PPARG coactivator 1 alpha	Transcription factor	10891
45	LGALS7B	galectin 7B		653499
46	IL17D	interleukin 17D		53342
47	LIN28B	lin-28 homolog B		389421
48	LDLR	low density lipoprotein receptor		3949
49	RCAN1	regulator of calcineurin 1	Signaling	1827
50	HSPA2	heat shock protein family A (Hsp70) member 2		3306
51	LGR5	leucine rich repeat containing G protein-coupled receptor 5	G-protein coupled receptor	8549
52	JAK2	Janus kinase 2	Kinase	3717
53	XAF1	XIAP associated factor 1		54739
54	B2M	beta-2-microglobulin	Immune response	567
55	KMT5A	lysine methyltransferase 5A		387893
56	EIF5A	eukaryotic translation initiation factor 5A	Nucleic acid binding	1984
57	MIR221	microRNA 221		407006
58	E2F4	E2F transcription factor 4	Nucleic acid binding	1874
59	CLDN10	claudin 10	Cell-cell junction	9071
60	TYMS	thymidylate synthetase	Enzyme	7298
61	ZFAS1	ZNFX1 antisense RNA 1		441951
62	SNCA	synuclein alpha	Transporter	6622
63	HRAS	HRas proto-oncogene, GTPase	Enzyme modulator	3265
64	FOLR2	folate receptor beta		2350
65	CDKN2B	cyclin dependent kinase inhibitor 2B		1030
66	VAT1	vesicle amine transport 1		10493
67	SOCS3	suppressor of cytokine signaling 3	Enzyme modulator	9021
68	KIF1B	kinesin family member 1B	Cellular structure	23095
69	MBD2	methyl-CpG binding domain protein 2	Epigenetic regulator	8932
70	FOXO1	forkhead box O1		2308
71	MAGI2-AS3	MAGI2 antisense RNA 3		1.01E+08
72	CKS2	CDC28 protein kinase regulatory subunit 2	Enzyme modulator	1164
73	RPS6KA2	ribosomal protein S6 kinase A2	Kinase	6196
74	MIR152	microRNA 152		406943
75	FPR1	formyl peptide receptor 1	G-protein coupled	2357

			receptor	
76	ADGRG1	adhesion G protein-coupled receptor G1	G-protein coupled receptor	9289
77	ANXA2	annexin A2		302
78	PDCD6	programmed cell death 6		10016
79	PRKN	parkin RBR E3 ubiquitin protein ligase	Enzyme	5071
80	F2RL2	coagulation factor II thrombin receptor like 2	G-protein coupled receptor	2151
81	LPAR3	lysophosphatidic acid receptor 3	G-protein coupled receptor	23566
82	SLC31A1	solute carrier family 31 member 1	Transporter	1317
83	ST7L	suppression of tumorigenicity 7 like		54879
84	IL10	interleukin 10		3586
85	PRMT5	protein arginine methyltransferase 5	Epigenetic regulator	10419
86	CLIC4	chloride intracellular channel 4	Ion channel	25932
87	KLK7	kallikrein related peptidase 7	Enzyme	5650
88	MDM2	MDM2 proto-oncogene	Nucleic acid binding	4193
89	NLK	nemo like kinase	Kinase	51701
90	SNHG15	small nucleolar RNA host gene 15		285958
91	CLDN3	claudin 3	Cell-cell junction	1365
92	AKT2	AKT serine/threonine kinase 2	Kinase	208
93	EGFR	epidermal growth factor receptor	Kinase	1956
94	GALNT6	polypeptide N-acetylgalactosaminyltransferase 6	Enzyme	11226
95	MIR130A	microRNA 130a		406919
96	PCGEM1	PCGEM1 prostate-specific transcript		64002
97	ERBB2	erb-b2 receptor tyrosine kinase 2	Kinase	2064
98	LGALS3	galectin 3	Signaling	3958
99	TMPRSS3	transmembrane serine protease 3	Enzyme	64699
100	SOHLH2	spermatogenesis and oogenesis specific basic helix-loop-helix 2		54937
101	MIR936	microRNA 936		1E+08
102	AKAP3	A-kinase anchoring protein 3		10566
103	TBL1XR1	TBL1X receptor 1		79718
104	TACSTD2	tumor associated calcium signal transducer 2		4070
105	NRG3	neuregulin 3	Signaling	10718
106	F10	coagulation factor X	Enzyme	2159
107	SRGAP1	SLIT-ROBO Rho GTPase activating protein 1	Enzyme modulator	57522
108	CCNE2	cyclin E2	Enzyme modulator	9134
109	ABHD8	abhydrolase domain containing 8		79575
110	RAD50	RAD50 double strand break repair protein		10111
111	SYNE1	spectrin repeat containing nuclear envelope protein 1		23345
112	ADH4	alcohol dehydrogenase 4 (class II), pi polypeptide	Enzyme	127
113	HTRA1	HtrA serine peptidase 1	Enzyme	5654
114	RAB22A	RAB22A, member RAS oncogene family		57403

115	EN2	engrailed homeobox 2	Transcription factor	2020
116	RUNX2	RUNX family transcription factor 2	Transcription factor	860
117	PGR	progesterone receptor	Nuclear receptor	5241
118	NR2F6	nuclear receptor subfamily 2 group F member 6	Nuclear receptor	2063
119	MIR22	microRNA 22		407004
120	BRMS1L	BRMS1 like transcriptional repressor		84312
121	ITCH	itchy E3 ubiquitin protein ligase	Enzyme	83737
122	TET1	tet methylcytosine dioxygenase 1		80312
123	DMD	dystrophin		1756
124	IGFBP3	insulin like growth factor binding protein 3	Enzyme modulator	3486
125	DEK	DEK proto-oncogene		7913
126	ARHGAP24	Rho GTPase activating protein 24		83478
127	C4BPA	complement component 4 binding protein alpha		722
128	ALB	albumin	Transporter	213
129	ACTBL2	actin beta like 2		345651
130	HLA-C	major histocompatibility complex, class I, C		3107
131	FZD4	frizzled class receptor 4	G-protein coupled receptor	8322
132	CASP4	caspase 4	Enzyme	837
133	MCPH1	microcephalin 1	Nucleic acid binding	79648
134	TWIST1	twist family bHLH transcription factor 1	Transcription factor	7291
135	MIR509-1	microRNA 509-1		574514
136	TYMP	thymidine phosphorylase	Enzyme	1890
137	SAFB	scaffold attachment factor B		6294
138	XRCC2	X-ray repair cross complementing 2		7516
139	MMS19	MMS19 homolog, cytosolic iron-sulfur assembly component	Transcription factor	64210
140	ERBB4	erb-b2 receptor tyrosine kinase 4	Kinase	2066
141	UGCG	UDP-glucose ceramide glucosyltransferase	Enzyme	7357
142	EIF6	eukaryotic translation initiation factor 6	Nucleic acid binding	3692
143	MIR146A	microRNA 146a		406938
144	KIF7	kinesin family member 7		374654
145	RAD51B	RAD51 paralog B		5890
146	MIR149	microRNA 149		406941
147	CD55	CD55 molecule (Cromer blood group)		1604
148	LNPEP	leucyl and cystinyl aminopeptidase	Enzyme	4012
149	MIR148A	microRNA 148a		406940
150	KRT13	keratin 13		3860
151	MIR204	microRNA 204		406987
152	EZH2	enhancer of zeste 2 polycomb repressive complex 2 subunit	Epigenetic regulator	2146
153	MIR127	microRNA 127		406914
154	PON1	paraoxonase 1		5444
155	HBEGF	heparin binding EGF like growth factor	Signaling	1839
156	MIR223	microRNA 223		407008
157	TIPARP	TCDD inducible poly(ADP-ribose) polymerase		25976

158	MIR502	microRNA 502		574504
159	CKB	creatine kinase B	Kinase	1152
160	GOLPH3	golgi phosphoprotein 3		64083
161	URI1	URI1 prefoldin like chaperone	Transcription factor	8725
162	CD40	CD40 molecule		958
163	MIR23A	microRNA 23a		407010
164	IGFBP7	insulin like growth factor binding protein 7		3490
165	MAL	mal, T cell differentiation protein	Transporter	4118
166	KIFC1	kinesin family member C1	Cellular structure	3833
167	ALDH1A2	aldehyde dehydrogenase 1 family member A2	Enzyme	8854
168	MIR574	microRNA 574		693159
169	EGR1	early growth response 1	Nucleic acid binding	1958
170	ABCB1	ATP binding cassette subfamily B member 1	Transporter	5243
171	KLF6	Kruppel like factor 6	Nucleic acid binding	1316
172	THBD	thrombomodulin		7056
173	UBR5	ubiquitin protein ligase E3 component n-recognin 5		51366
174	MAPK14	mitogen-activated protein kinase 14	Kinase	1432
175	SERPINB2	serpin family B member 2	Enzyme modulator	5055
176	MIR106B	microRNA 106b		406900
177	CD226	CD226 molecule		10666
178	STAT3	signal transducer and activator of transcription 3	Nucleic acid binding	6774
179	MAD1L1	mitotic arrest deficient 1 like 1		8379
180	CAP1	cyclase associated actin cytoskeleton regulatory protein 1	Cellular structure	10487
181	HSPA1A	heat shock protein family A (Hsp70) member 1A		3303
182	ARRB1	arrestin beta 1	Enzyme modulator	408
183	DAPK2	death associated protein kinase 2	Kinase	23604
184	CENPA	centromere protein A		1058
185	MCAM	melanoma cell adhesion molecule	Cell adhesion	4162
186	SFN	stratifin	Chaperone	2810
187	TMED2	transmembrane p24 trafficking protein 2	Transporter	10959
188	ANG	angiogenin		283
189	CASC2	cancer susceptibility 2		255082
190	KEAP1	kelch like ECH associated protein 1		9817
191	HOXA7	homeobox A7	Transcription factor	3204
192	SERPIND1	serpin family D member 1	Enzyme modulator	3053
193	MARCHF7	membrane associated ring-CH-type finger 7		64844
194	IL27	interleukin 27		246778
195	KLK15	kallikrein related peptidase 15	Enzyme	55554
196	GABPA	GA binding protein transcription factor subunit alpha	Transcription factor	2551
197	CASP3	caspase 3	Enzyme	836
198	ITGAM	integrin subunit alpha M		3684
199	DIRAS3	DIRAS family GTPase 3	Enzyme modulator	9077
200	SNHG20	small nucleolar RNA host gene 20		654434

201	PECAM1	platelet and endothelial cell adhesion molecule 1	Cell adhesion	5175
202	LOC110806263	TERT 5' regulatory region		1.11E+08
203	SKAP1	src kinase associated phosphoprotein 1		8631
204	DSC3	desmocollin 3	Cell adhesion	1825
205	LHCGR	luteinizing hormone/choriogonadotropin receptor	G-protein coupled receptor	3973
206	CCR1	C-C motif chemokine receptor 1	G-protein coupled receptor	1230
207	DUSP6	dual specificity phosphatase 6		1848
208	ELK1	ETS transcription factor ELK1	Transcription factor	2002
209	LGMN	legumain	Enzyme	5641
210	LILRB1	leukocyte immunoglobulin like receptor B1	Receptor	10859
211	NFIX	nuclear factor I X	Nucleic acid binding	4784
212	CLEC14A	C-type lectin domain containing 14A		161198
213	LPA	lipoprotein(a)	Enzyme	4018
214	LY75	lymphocyte antigen 75		4065
215	NQO1	NAD(P)H quinone dehydrogenase 1		1728
216	SP1	Sp1 transcription factor	Nucleic acid binding	6667
217	GTF2F1	general transcription factor IIF subunit 1	Nucleic acid binding	2962
218	IGFBP1	insulin like growth factor binding protein 1	Enzyme modulator	3484
219	IFNG	interferon gamma		3458
220	HTATIP2	HIV-1 Tat interactive protein 2		10553
221	FGF2	fibroblast growth factor 2	Signaling	2247
222	ING4	inhibitor of growth family member 4	Nucleic acid binding	51147
223	RRM2	ribonucleotide reductase regulatory subunit M2	Enzyme	6241
224	MRE11	MRE11 homolog, double strand break repair nuclease		4361
225	MIR429	microRNA 429		554210
226	KLRC4-KLRK1	KLRC4-KLRK1 readthrough		1.01E+08
227	KLK5	kallikrein related peptidase 5	Enzyme	25818
228	NOB1	NIN1 (RPN12) binding protein 1 homolog		28987
229	TSG101	tumor susceptibility 101	Enzyme	7251
230	TNFRSF1B	TNF receptor superfamily member 1B		7133
231	CXCL1	C-X-C motif chemokine ligand 1	Signaling	2919
232	PAX8	paired box 8		7849
233	PRDX2	peroxiredoxin 2	Enzyme	7001
234	CDKN1C	cyclin dependent kinase inhibitor 1C		1028
235	RBMS3	RNA binding motif single stranded interacting protein 3		27303
236	TGFBR2	transforming growth factor beta receptor 2	Kinase	7048
237	IL33	interleukin 33		90865
238	MSLN	mesothelin	Extracellular structure	10232
239	WWTR1	WW domain containing transcription regulator 1	Enzyme modulator	25937

240	CIB1	calcium and integrin binding 1		10519
241	SETD7	SET domain containing 7, histone lysine methyltransferase	Epigenetic regulator	80854
242	MIRLET7A3	microRNA let-7a-3		406883
243	VEGFD	vascular endothelial growth factor D	Signaling	2277
244	HDGF	heparin binding growth factor	Signaling	3068
245	PRAME	preferentially expressed antigen in melanoma		23532
246	TRIM59	tripartite motif containing 59		286827
247	PPA1	inorganic pyrophosphatase 1	Enzyme	5464
248	NOTCH3	notch receptor 3		4854
249	CDKN3	cyclin dependent kinase inhibitor 3		1033
250	SFRP1	secreted frizzled related protein 1		6422
251	RASSF1	Ras association domain family member 1	Enzyme modulator	11186
252	HOTTIP	HOXA distal transcript antisense RNA		1E+08
253	BTG3	BTG anti-proliferation factor 3		10950
254	FBLN5	fibulin 5	Calcium-binding protein	10516
255	MIR490	microRNA 490		574443
256	PMS2	PMS1 homolog 2, mismatch repair system component	Nucleic acid binding	5395
257	HELQ	helicase, POLQ like		113510
258	HOXB-AS3	HOXB cluster antisense RNA 3		404266
259	WASF1	WASP family member 1	Cellular structure	8936
260	HSPB1	heat shock protein family B (small) member 1		3315
261	IGF1	insulin like growth factor 1		3479
262	GDF15	growth differentiation factor 15	Signaling	9518
263	MIR545	microRNA 545		664614
264	DKK2	dickkopf WNT signaling pathway inhibitor 2		27123
265	ZIC2	Zic family member 2	Transcription factor	7546
266	KPNA2	karyopherin subunit alpha 2	Transporter	3838
267	PDIA3	protein disulfide isomerase family A member 3		2923
268	NECTIN2	nectin cell adhesion molecule 2		5819
269	GNAS	GNAS complex locus	Enzyme modulator	2778
270	MIR302B	microRNA 302b		442894
271	MIR520H	microRNA 520h		574493
272	PIN1	peptidylprolyl cis/trans isomerase, NIMA-interacting 1		5300
273	MIR99A	microRNA 99a		407055
274	TRIM2	tripartite motif containing 2	Enzyme	23321
275	HYOU1	hypoxia up-regulated 1		10525
276	DLX6-AS1	DLX6 antisense RNA 1		285987
277	USP7	ubiquitin specific peptidase 7	Enzyme	7874
278	KL	klotho	Enzyme	9365
279	RACGAP1	Rac GTPase activating protein 1	Enzyme modulator	29127
280	EDNRA	endothelin receptor type A	G-protein coupled receptor	1909

281	ESRRA	estrogen related receptor alpha	Nuclear receptor	2101
282	BAGE	B melanoma antigen		574
283	SLC22A16	solute carrier family 22 member 16	Transporter	85413
284	CRP	C-reactive protein		1401
285	MED12	mediator complex subunit 12	Transcription factor	9968
286	RABEP2	rabaptin, RAB GTPase binding effector protein 2		79874
287	FUBP1	far upstream element binding protein 1	Enzyme	8880
288	TNFSF12	TNF superfamily member 12		8742
289	MIR211	microRNA 211		406993
290	YWHAZ	tyrosine 3-monooxygenase/tryptophan 5-monooxygenase activation protein zeta	Chaperone	7534
291	RPL10	ribosomal protein L10	Nucleic acid binding	6134
292	CARD14	caspase recruitment domain family member 14		79092
293	NCOA3	nuclear receptor coactivator 3	Kinase	8202
294	CCAT1	colon cancer associated transcript 1		1.01E+08
295	TPT1	tumor protein, translationally-controlled 1	Cellular structure	7178
296	CEBPD	CCAAT enhancer binding protein delta	Transcription factor	1052
297	CTSB	cathepsin B	Enzyme	1508
298	TNF	tumor necrosis factor	Signaling	7124
299	IL1A	interleukin 1 alpha		3552
300	PDGFD	platelet derived growth factor D	Signaling	80310
301	ERCC1	ERCC excision repair 1, endonuclease non-catalytic subunit	Enzyme	2067
302	PAX1	paired box 1		5075
303	MAP1LC3B	microtubule associated protein 1 light chain 3 beta	Cellular structure	81631
304	UCA1	urothelial cancer associated 1		652995
305	MIR542	microRNA 542		664617
306	SPP1	secreted phosphoprotein 1		6696
307	GLI1	GLI family zinc finger 1		2735
308	PTGS2	prostaglandin-endoperoxide synthase 2	Enzyme	5743
309	HPGDS	hematopoietic prostaglandin D synthase		27306
310	CTSK	cathepsin K	Enzyme	1513
311	PSIP1	PC4 and SFRS1 interacting protein 1	Signaling	11168
312	BMP2	bone morphogenetic protein 2	Signaling	650
313	ESR1	estrogen receptor 1	Nuclear receptor	2099
314	SST	somatostatin	Signaling	6750
315	CDK4	cyclin dependent kinase 4	Kinase	1019
316	RBM3	RNA binding motif protein 3	Nucleic acid binding	5935
317	PIK3CD	phosphatidylinositol-4,5-bisphosphate 3-kinase catalytic subunit delta	Kinase	5293
318	SLC7A11	solute carrier family 7 member 11	Transporter	23657
319	SPATA2	spermatogenesis associated 2		9825
320	WWOX	WW domain containing oxidoreductase	Enzyme	51741
321	FUT8	fucosyltransferase 8		2530
322	STAT1	signal transducer and activator of transcription 1	Nucleic acid binding	6772

323	CHMP4C	charged multivesicular body protein 4C	Transporter	92421
324	CD68	CD68 molecule	Transporter	968
325	MIR338	microRNA 338		442906
326	BSG	basigin (Ok blood group)		682
327	LINC01088	long intergenic non-protein coding RNA 1088		1.01E+08
328	MIR335	microRNA 335		442904
329	HACD1	3-hydroxyacyl-CoA dehydratase 1		9200
330	MIR134	microRNA 134		406924
331	CHGB	chromogranin B		1114
332	DIAPH3	diaphanous related formin 3		81624
333	COL4A2	collagen type IV alpha 2 chain		1284
334	COL11A2	collagen type XI alpha 2 chain		1302
335	LDLRAP1	low density lipoprotein receptor adaptor protein 1	Signaling	26119
336	ANGPTL4	angiopoietin like 4	Signaling	51129
337	LGALS7	galectin 7	Signaling	3963
338	MAPK1	mitogen-activated protein kinase 1	Kinase	5594
339	EIF3A	eukaryotic translation initiation factor 3 subunit A	Nucleic acid binding	8661
340	IKBKB	inhibitor of nuclear factor kappa B kinase subunit beta	Kinase	3551
341	DOK1	docking protein 1		1796
342	HOXD10	homeobox D10		3236
343	FSCN1	fascin actin-bundling protein 1	Cellular structure	6624
344	BRD7	bromodomain containing 7	Epigenetic regulator	29117
345	PRSS55	serine protease 55	Enzyme	203074
346	OPCML	opioid binding protein/cell adhesion molecule like		4978
347	MAPK8	mitogen-activated protein kinase 8	Kinase	5599
348	SYTL2	synaptotagmin like 2		54843
349	PSMB4	proteasome 20S subunit beta 4		5692
350	MIR1294	microRNA 1294		1E+08
351	SUZ12	SUZ12 polycomb repressive complex 2 subunit	Nucleic acid binding	23512
352	MACC1	MET transcriptional regulator MACC1		346389
353	TRIM28	tripartite motif containing 28		10155
354	WFDC2	WAP four-disulfide core domain 2	Enzyme modulator	10406
355	MIR25	microRNA 25		407014
356	IL1RN	interleukin 1 receptor antagonist		3557
357	ADIPOR1	adiponectin receptor 1	Receptor	51094
358	CYP17A1	cytochrome P450 family 17 subfamily A member 1		1586
359	CCL5	C-C motif chemokine ligand 5	Signaling	6352
360	MIR373	microRNA 373		442918
361	LOX	lysyl oxidase		4015
362	MIR455	microRNA 455		619556
363	BAG5	BAG cochaperone 5		9529

364	CCN1	cellular communication network factor 1	Signaling	3491
365	PDCD1	programmed cell death 1		5133
366	MAP3K7	mitogen-activated protein kinase kinase kinase 7	Kinase	6885
367	BAG4	BAG cochaperone 4		9530
368	CAT	catalase	Enzyme	847
369	ABCA2	ATP binding cassette subfamily A member 2	Transporter	20
370	ADM	adrenomedullin	Signaling	133
371	ANKRD30A	ankyrin repeat domain 30A		91074
372	CD47	CD47 molecule		961
373	POU5F1	POU class 5 homeobox 1		5460
374	NFKB1	nuclear factor kappa B subunit 1	Transcription factor	4790
375	MUC13	mucin 13, cell surface associated		56667
376	IL21	interleukin 21		59067
377	TUBA4B	tubulin alpha 4b	Cellular structure	80086
378	GALNT1	polypeptide N-acetylgalactosaminyltransferase 1	Enzyme	2589
379	HGFAC	HGF activator	Enzyme	3083
380	EPHB2	EPH receptor B2	Kinase	2048
381	HAVCR2	hepatitis A virus cellular receptor 2		84868
382	PLAUR	plasminogen activator, urokinase receptor		5329
383	NME1	NME/NM23 nucleoside diphosphate kinase 1	Kinase	4830
384	HGS	hepatocyte growth factor-regulated tyrosine kinase substrate		9146
385	SLC16A1	solute carrier family 16 member 1	Transporter	6566
386	PYCARD	PYD and CARD domain containing	Enzyme	29108
387	SOX2	SRY-box transcription factor 2	Transcription factor	6657
388	COMT	catechol-O-methyltransferase	Enzyme	1312
389	PDLIM5	PDZ and LIM domain 5	Cellular structure	10611
390	LSINCT5	long stress-induced non-coding transcript 5		1.01E+08
391	SPINK1	serine peptidase inhibitor Kazal type 1		6690
392	AAVS1	adeno-associated virus integration site 1		17
393	NBN	nibrin	Nucleic acid binding	4683
394	BTLA	B and T lymphocyte associated		151888
395	TUG1	taurine up-regulated 1		55000
396	SYNPO2	synaptopodin 2	Cellular structure	171024
397	NRP1	neuropilin 1		8829
398	CLDN4	claudin 4	Cell-cell junction	1364
399	SLC22A18	solute carrier family 22 member 18	Transporter	5002
400	ERLNC1	estrogen receptor responsive lncRNA 1		1.02E+08
401	EPHA2	EPH receptor A2	Kinase	1969
402	MIR18A	microRNA 18a		406953
403	CARD11	caspase recruitment domain family member 11		84433
404	ST3GAL1	ST3 beta-galactoside alpha-2,3-sialyltransferase 1		6482
405	DICER1	dicer 1, ribonuclease III	Enzyme	23405
406	LMLN	leishmanolysin like peptidase	Enzyme	89782
407	BDNF	brain derived neurotrophic factor	Signaling	627
408	CSF1	colony stimulating factor 1		1435

409	CAMK2D	calcium/calmodulin dependent protein kinase II delta	Kinase	817
410	E2F1	E2F transcription factor 1	Nucleic acid binding	1869
411	NRP2	neuropilin 2		8828
412	LAG3	lymphocyte activating 3	Receptor	3902
413	KRAS	KRAS proto-oncogene, GTPase	Enzyme modulator	3845
414	CDX2	caudal type homeobox 2	Transcription factor	1045
415	RPSA	ribosomal protein SA	Nucleic acid binding	3921
416	MAPT	microtubule associated protein tau		4137
417	CTAG2	cancer/testis antigen 2		30848
418	CXCL8	C-X-C motif chemokine ligand 8	Signaling	3576
419	ECM1	extracellular matrix protein 1		1893
420	EGFL7	EGF like domain multiple 7	Calcium-binding protein	51162
421	YY1	YY1 transcription factor	Transcription factor	7528
422	CCN2	cellular communication network factor 2	Signaling	1490
423	CDK6	cyclin dependent kinase 6	Kinase	1021
424	ANO1	anoctamin 1	Ion channel	55107
425	SMAD7	SMAD family member 7	Transcription factor	4092
426	FGFR2	fibroblast growth factor receptor 2	Kinase	2263
427	XRCC3	X-ray repair cross complementing 3		7517
428	CSF3	colony stimulating factor 3		1440
429	ACRBP	acrosin binding protein		84519
430	CHUK	component of inhibitor of nuclear factor kappa B kinase complex	Kinase	1147
431	IL22	interleukin 22		50616
432	MIR506	microRNA 506		574511
433	RAD51C	RAD51 paralog C		5889
434	HAGLROS	HAGLR opposite strand lncRNA		1.03E+08
435	SFRP4	secreted frizzled related protein 4		6424
436	AGTR1	angiotensin II receptor type 1	G-protein coupled receptor	185
437	RRBP1	ribosome binding protein 1		6238
438	CSNK1D	casein kinase 1 delta	Kinase	1453
439	PRSS3	serine protease 3	Enzyme	5646
440	CTSD	cathepsin D	Enzyme	1509
441	MIR510	microRNA 510		574515
442	TWNK	twinkle mtDNA helicase	Enzyme	56652
443	PTCH1	patched 1		5727
444	ERCC3	ERCC excision repair 3, TFIIH core complex helicase subunit	Enzyme	2071
445	SERPINF2	serpin family F member 2	Enzyme modulator	5345
446	FANCF	FA complementation group F		2188
447	CHKA	choline kinase alpha	Kinase	1119
448	HSP90AA1	heat shock protein 90 alpha family class A member 1	Chaperone	3320

449	PARP1	poly(ADP-ribose) polymerase 1		142
450	CD34	CD34 molecule		947
451	MCTS1	MCTS1 re-initiation and release factor	Receptor	28985
452	GPX6	glutathione peroxidase 6	Enzyme	257202
453	ENG	endoglin		2022
454	SULT1E1	sulfotransferase family 1E member 1		6783
455	MYLIP	myosin regulatory light chain interacting protein		29116
456	STN1	STN1 subunit of CST complex		79991
457	CARM1	coactivator associated arginine methyltransferase 1	Epigenetic regulator	10498
458	SFRP5	secreted frizzled related protein 5		6425
459	MYD88	MYD88 innate immune signal transduction adaptor	Enzyme modulator	4615
460	HOXA11-AS	HOXA11 antisense RNA		221883
461	EBAG9	estrogen receptor binding site associated antigen 9		9166
462	ASPM	abnormal spindle microtubule assembly		259266
463	RACK1	receptor for activated C kinase 1		10399
464	IL2RA	interleukin 2 receptor subunit alpha	Receptor	3559
465	CD24	CD24 molecule		1E+08
466	NSF	N-ethylmaleimide sensitive factor, vesicle fusing ATPase	Enzyme	4905
467	FAP	fibroblast activation protein alpha	Enzyme	2191
468	MIR802	microRNA 802		768219
469	LINC01118	long intergenic non-protein coding RNA 1118		388948
470	SERPINA7	serpin family A member 7	Enzyme modulator	6906
471	DENR	density regulated re-initiation and release factor	Enzyme	8562
472	ADRA2B	adrenoceptor alpha 2B	G-protein coupled receptor	151
473	PEA15	proliferation and apoptosis adaptor protein 15		8682
474	FGF7	fibroblast growth factor 7	Signaling	2252
475	IL37	interleukin 37		27178
476	GSTK1	glutathione S-transferase kappa 1		373156
477	FOXA1	forkhead box A1	Transcription factor	3169
478	ABI1	abl interactor 1	Enzyme modulator	10006
479	AMH	anti-Mullerian hormone		268
480	TET2	tet methylcytosine dioxygenase 2		54790
481	HK2	hexokinase 2	Kinase	3099
482	EPHX1	epoxide hydrolase 1		2052
483	TNFAIP8	TNF alpha induced protein 8		25816
484	ICAM1	intercellular adhesion molecule 1		3383
485	CALCRL	calcitonin receptor like receptor	G-protein coupled receptor	10203
486	BCL2L1	BCL2 like 1	Signaling	598
487	MIRLET7I	microRNA let-7i		406891

488	MIR136	microRNA 136		406927
489	MAPK3	mitogen-activated protein kinase 3	Kinase	5595
490	MIR940	microRNA 940		1E+08
491	TRIM44	tripartite motif containing 44		54765
492	GRN	granulin precursor		2896
493	PROM1	prominin 1	Transporter	8842
494	MIR10B	microRNA 10b		406903
495	STOML2	stomatin like 2	Enzyme modulator	30968
496	MSH2	mutS homolog 2	Nucleic acid binding	4436
497	ZEB2	zinc finger E-box binding homeobox 2	Transcription factor	9839
498	CYLD	CYLD lysine 63 deubiquitinase	Enzyme	1540
499	SERPINE1	serpin family E member 1	Enzyme modulator	5054
500	HFE	homeostatic iron regulator		3077
501	SMN1	survival of motor neuron 1, telomeric	Epigenetic regulator	6606
502	SLC13A5	solute carrier family 13 member 5	Transporter	284111
503	BACH1	BTB domain and CNC homolog 1		571
504	RSAD2	radical S-adenosyl methionine domain containing 2		91543
505	CTBP2	C-terminal binding protein 2	Transcription factor	1488
506	USHBP1	USH1 protein network component harmonin binding protein 1		83878
507	MEG3	maternally expressed 3		55384
508	RAD51	RAD51 recombinase		5888
509	CRK	CRK proto-oncogene, adaptor protein		1398
510	RNASET2	ribonuclease T2		8635
511	NEDD9	neural precursor cell expressed, developmentally down-regulated 9		4739
512	HSPA1B	heat shock protein family A (Hsp70) member 1B		3304
513	ETV5	ETS variant transcription factor 5	Transcription factor	2119
514	SLC39A11	solute carrier family 39 member 11	Transporter	201266
515	CCND2	cyclin D2	Enzyme modulator	894
516	CDCP1	CUB domain containing protein 1		64866
517	PAX3	paired box 3		5077
518	KIF20A	kinesin family member 20A	Cellular structure	10112
519	YAP1	Yes associated protein 1	Enzyme modulator	10413
520	SIK3	SIK family kinase 3	Kinase	23387
521	ITGB3	integrin subunit beta 3	Receptor	3690
522	BCAT1	branched chain amino acid transaminase 1	Enzyme	586
523	KLK11	kallikrein related peptidase 11	Enzyme	11012
524	HMMR-AS1	HMMR antisense RNA 1		1.02E+08
525	PARD3	par-3 family cell polarity regulator		56288
526	TGFBI	transforming growth factor beta induced	Signaling	7045
527	HSPG2	heparan sulfate proteoglycan 2	Enzyme modulator	3339
528	CD274	CD274 molecule	Receptor	29126
529	SLC52A2	solute carrier family 52 member 2	Transporter	79581
530	TRIB2	tribbles pseudokinase 2	Kinase	28951

531	MAD2L2	mitotic arrest deficient 2 like 2		10459
532	ZNF300P1	zinc finger protein 300 pseudogene 1		134466
533	GHRH	growth hormone releasing hormone	Signaling	2691
534	ADIPOQ	adiponectin, C1Q and collagen domain containing		9370
535	CD44	CD44 molecule (Indian blood group)		960
536	DPY30	dpy-30 histone methyltransferase complex regulatory subunit		84661
537	GALNT3	polypeptide N-acetylgalactosaminyltransferase 3	Enzyme	2591
538	NTRK1	neurotrophic receptor tyrosine kinase 1	Kinase	4914
539	CCNB1	cyclin B1	Enzyme modulator	891
540	MET	MET proto-oncogene, receptor tyrosine kinase	Kinase	4233
541	LEP	leptin		3952
542	CCL22	C-C motif chemokine ligand 22	Signaling	6367
543	CYP2D6	cytochrome P450 family 2 subfamily D member 6		1565
544	RUNX3	RUNX family transcription factor 3	Transcription factor	864
545	ELANE	elastase, neutrophil expressed	Enzyme	1991
546	VGLL3	vestigial like family member 3		389136
547	DNAJC15	DnaJ heat shock protein family (Hsp40) member C15		29103
548	CCNE1	cyclin E1	Enzyme modulator	898
549	HSPD1	heat shock protein family D (Hsp60) member 1		3329
550	SHBG	sex hormone binding globulin		6462
551	MIR200C	microRNA 200c		406985
552	BCL2	BCL2 apoptosis regulator	Signaling	596
553	KCNMA1-AS1	KCNMA1 antisense RNA 1		1.02E+08
554	TOMM34	translocase of outer mitochondrial membrane 34		10953
555	GSTM4	glutathione S-transferase mu 4		2948
556	DPYD	dihydropyrimidine dehydrogenase	Enzyme	1806
557	KDM2A	lysine demethylase 2A		22992
558	AGT	angiotensinogen	Enzyme modulator	183
559	IL6R	interleukin 6 receptor	Signaling	3570
560	MNX1-AS1	MNX1 antisense RNA 1 (head to head)		645249
561	NQO2	N-ribosyldihydronicotinamide:quinone reductase 2		4835
562	NEU1	neuraminidase 1	Enzyme	4758
563	MAK16	MAK16 homolog		84549
564	SPRY2	sprouty RTK signaling antagonist 2	Signaling	10253
565	XPC	XPC complex subunit, DNA damage recognition and repair factor	Nucleic acid binding	7508
566	NFYA	nuclear transcription factor Y subunit alpha	Nucleic acid binding	4800
567	KIF14	kinesin family member 14	Cellular structure	9928
568	NFE2L2	nuclear factor, erythroid 2 like 2	Enzyme	4780
569	NRF1	nuclear respiratory factor 1		4899
570	TBX21	T-box transcription factor 21	Transcription factor	30009

571	CCT4	chaperonin containing TCP1 subunit 4	Chaperone	10575
572	MIR199A2	microRNA 199a-2		406977
573	BRD4	bromodomain containing 4	Epigenetic regulator	23476
574	CYP19A1	cytochrome P450 family 19 subfamily A member 1	Enzyme	1588
575	MIR29B2	microRNA 29b-2		407025
576	ELAVL2	ELAV like RNA binding protein 2		1993
577	SET	SET nuclear proto-oncogene	Enzyme modulator	6418
578	NKX2-8	NK2 homeobox 8	Transcription factor	26257
579	XIAP	X-linked inhibitor of apoptosis	Enzyme modulator	331
580	CA9	carbonic anhydrase 9		768
581	NF1	neurofibromin 1	Enzyme modulator	4763
582	MIRLET7E	microRNA let-7e		406887
583	AGER	advanced glycosylation end-product specific receptor	Receptor	177
584	MICA	MHC class I polypeptide-related sequence A		1.01E+08
585	LINC01194	long intergenic non-protein coding RNA 1194		404663
586	NT5E	5'-nucleotidase ecto	Enzyme	4907
587	IL18	interleukin 18		3606
588	IGFBP2	insulin like growth factor binding protein 2	Enzyme modulator	3485
589	KDM6B	lysine demethylase 6B	Epigenetic regulator	23135
590	ACLY	ATP citrate lyase	Enzyme	47
591	CSNK2A2	casein kinase 2 alpha 2	Kinase	1459
592	MELK	maternal embryonic leucine zipper kinase	Kinase	9833
593	ITGB2	integrin subunit beta 2	Receptor	3689
594	CTCFL	CCCTC-binding factor like	Transcription factor	140690
595	VEGFA	vascular endothelial growth factor A	Signaling	7422
596	XRCC1	X-ray repair cross complementing 1		7515
597	SENP3	SUMO specific peptidase 3	Enzyme	26168
598	MYC	MYC proto-oncogene, bHLH transcription factor	Transcription factor	4609
599	SPDYA	speedy/RINGO cell cycle regulator family member A		245711
600	MUS81	MUS81 structure-specific endonuclease subunit		80198
601	SRSF3	serine and arginine rich splicing factor 3	Nucleic acid binding	6428
602	MIR21	microRNA 21		406991
603	ATG3	autophagy related 3	Enzyme	64422
604	MMP1	matrix metallopeptidase 1	Enzyme	4312
605	GCLC	glutamate-cysteine ligase catalytic subunit	Enzyme	2729
606	MIR539	microRNA 539		664612
607	PES1	pescadillo ribosomal biogenesis factor 1	Nucleic acid binding	23481
608	TBC1D16	TBC1 domain family member 16	Enzyme	125058
609	MIR3129	microRNA 3129		1E+08
610	IDO1	indoleamine 2,3-dioxygenase 1		3620
611	HOXD8	homeobox D8		3234
612	MMP3	matrix metallopeptidase 3	Enzyme	4314
613	MIR590	microRNA 590		693175

614	CCL18	C-C motif chemokine ligand 18	Signaling	6362
615	BRCA2	BRCA2 DNA repair associated	Nucleic acid binding	675
616	FMR1	FMRP translational regulator 1	Nucleic acid binding	2332
617	ERCC4	ERCC excision repair 4, endonuclease catalytic subunit	Enzyme	2072
618	MIR34A	microRNA 34a		407040
619	MIR222	microRNA 222		407007
620	ANXA10	annexin A10		11199
621	MIR532	microRNA 532		693124
622	SPRY1	sprouty RTK signaling antagonist 1	Signaling	10252
623	MMP14	matrix metallopeptidase 14	Enzyme	4323
624	SOD1	superoxide dismutase 1	Enzyme	6647
625	DIABLO	diablo IAP-binding mitochondrial protein		56616
626	CLPTM1L	CLPTM1 like	Enzyme	81037
627	THBS1	thrombospondin 1		7057
628	GPER1	G protein-coupled estrogen receptor 1	G-protein coupled receptor	2852
629	MYDGF	myeloid derived growth factor		56005
630	CDK7	cyclin dependent kinase 7	Kinase	1022
631	CHEK2	checkpoint kinase 2	Kinase	11200
632	MCM2	minichromosome maintenance complex component 2	Enzyme	4171
633	MIR155	microRNA 155		406947
634	SYF2	SYF2 pre-mRNA splicing factor		25949
635	PTPN6	protein tyrosine phosphatase non-receptor type 6		5777
636	MTA1	metastasis associated 1	Nucleic acid binding	9112
637	CDK2	cyclin dependent kinase 2	Kinase	1017
638	INTS2	integrator complex subunit 2		57508
639	MIR219A1	microRNA 219a-1		407002
640	MECOM	MDS1 and EVI1 complex locus		2122
641	ESRP1	epithelial splicing regulatory protein 1	Nucleic acid binding	54845
642	GALT	galactose-1-phosphate uridylyltransferase	Enzyme	2592
643	TPT1-AS1	TPT1 antisense RNA 1		1E+08
644	VCAN	versican	Extracellular structure	1462
645	RNH1	ribonuclease/angiogenin inhibitor 1	Transcription factor	6050
646	ALKBH5	alkB homolog 5, RNA demethylase		54890
647	FGF13	fibroblast growth factor 13	Signaling	2258
648	CDKN1A	cyclin dependent kinase inhibitor 1A		1026
649	LSR	lipolysis stimulated lipoprotein receptor		51599
650	GRP	gastrin releasing peptide		2922
651	CSTB	cystatin B	Enzyme modulator	1476
652	FPR2	formyl peptide receptor 2	G-protein coupled receptor	2358
653	BMI1	BMI1 proto-oncogene, polycomb ring finger		648
654	FOSB	FosB proto-oncogene, AP-1 transcription factor	Transcription factor	2354

		subunit		
655	S100A7	S100 calcium binding protein A7	Calcium-binding protein	6278
656	CXCR6	C-X-C motif chemokine receptor 6	G-protein coupled receptor	10663
657	HPSE	heparanase	Enzyme	10855
658	NFKBIA	NFKB inhibitor alpha		4792
659	MLH1	mutL homolog 1	Nucleic acid binding	4292
660	KISS1	KiSS-1 metastasis suppressor		3814
661	SERPINB3	serpin family B member 3	Enzyme modulator	6317
662	LIN28A	lin-28 homolog A	Nucleic acid binding	79727
663	MARCHF1	membrane associated ring-CH-type finger 1		55016
664	BNC2	basonuclin 2	Nucleic acid binding	54796
665	ROR1	receptor tyrosine kinase like orphan receptor 1	Kinase	4919
666	BRAF	B-Raf proto-oncogene, serine/threonine kinase	Kinase	673
667	BABAM2	BRISC and BRCA1 A complex member 2		9577
668	AKT3	AKT serine/threonine kinase 3	Kinase	10000
669	AMHR2	anti-Mullerian hormone receptor type 2	Kinase	269
670	LINC00472	long intergenic non-protein coding RNA 472		79940
671	ACKR3	atypical chemokine receptor 3	G-protein coupled receptor	57007
672	HYAL1	hyaluronidase 1	Enzyme	3373
673	AGR2	anterior gradient 2, protein disulphide isomerase family member	Enzyme	10551
674	HMGB1	high mobility group box 1	Nucleic acid binding	3146
675	DPH1	diphthamide biosynthesis 1		1801
676	ARHGEF10L	Rho guanine nucleotide exchange factor 10 like	Enzyme	55160
677	PTN	pleiotrophin	Signaling	5764
678	RYK	receptor like tyrosine kinase	Kinase	6259
679	SOX7	SRY-box transcription factor 7	Transcription factor	83595
680	KLK6	kallikrein related peptidase 6	Enzyme	5653
681	RECQL	RecQ like helicase	Enzyme	5965
682	SOX4	SRY-box transcription factor 4	Transcription factor	6659
683	CIB2	calcium and integrin binding family member 2		10518
684	MAML2	mastermind like transcriptional coactivator 2	Transcription factor	84441
685	MIR98	microRNA 98		407054
686	NFATC1	nuclear factor of activated T cells 1	Transcription factor	4772
687	PLCG1	phospholipase C gamma 1	Enzyme	5335
688	UBE2I	ubiquitin conjugating enzyme E2 I		7329
689	MUC16	mucin 16, cell surface associated		94025
690	FAS	Fas cell surface death receptor		355
691	BTN3A2	butyrophilin subfamily 3 member A2	Enzyme modulator	11118
692	ARF4	ADP ribosylation factor 4		378
693	MIR215	microRNA 215		406997
694	MIR320A	microRNA 320a		407037

695	BCL9	BCL9 transcription coactivator		607
696	ABO	ABO, alpha 1-3-N-acetylgalactosaminyltransferase and alpha 1-3-galactosyltransferase	Enzyme	28
697	USP10	ubiquitin specific peptidase 10	Enzyme	9100
698	F9	coagulation factor IX	Enzyme	2158
699	STAT4	signal transducer and activator of transcription 4	Nucleic acid binding	6775
700	PRDX1	peroxiredoxin 1	Enzyme	5052
701	LINC01116	long intergenic non-protein coding RNA 1116		375295
702	PIWIL1	piwi like RNA-mediated gene silencing 1		9271
703	FOLR1	folate receptor alpha		2348
704	TCF12	transcription factor 12	Transcription factor	6938
705	ALOX15	arachidonate 15-lipoxygenase	Enzyme	246
706	TP73	tumor protein p73	Transcription factor	7161
707	DSC2	desmocollin 2	Cell adhesion	1824
708	RET	ret proto-oncogene	Kinase	5979
709	RUNX1	RUNX family transcription factor 1	Transcription factor	861
710	CLDN7	claudin 7	Cell-cell junction	1366
711	STON2	stonin 2	Extracellular structure	85439
712	GRHL2	grainyhead like transcription factor 2	Transcription factor	79977
713	FAM215A	family with sequence similarity 215 member A		23591
714	ABCC4	ATP binding cassette subfamily C member 4	Transporter	10257
715	CDKN2D	cyclin dependent kinase inhibitor 2D		1032
716	STUB1	STIP1 homology and U-box containing protein 1		10273
717	FBXW12	F-box and WD repeat domain containing 12		285231
718	WWC1	WW and C2 domain containing 1		23286
719	MIR363	microRNA 363		574031
720	EFNA1	ephrin A1	Kinase	1942
721	RIF1	replication timing regulatory factor 1		55183
722	VEGFC	vascular endothelial growth factor C	Signaling	7424
723	LARP1	La ribonucleoprotein 1, translational regulator	Nucleic acid binding	23367
724	INVS	inversin		27130
725	MPO	myeloperoxidase	Enzyme	4353
726	VIM	vimentin		7431
727	HOXA10	homeobox A10		3206
728	NR1H2	nuclear receptor subfamily 1 group H member 2	Nuclear receptor	7376
729	RUVBL1	RuvB like AAA ATPase 1		8607
730	DUSP1	dual specificity phosphatase 1		1843
731	TRIM27	tripartite motif containing 27		5987
732	H19	H19 imprinted maternally expressed transcript		283120
733	TGFBR1	transforming growth factor beta receptor 1	Kinase	7046
734	ACVR1C	activin A receptor type 1C	Kinase	130399
735	ATP1B2	ATPase Na+/K+ transporting subunit beta 2	Transporter	482
736	ATM	ATM serine/threonine kinase	Kinase	472
737	MTERF1	mitochondrial transcription termination factor 1		7978

738	SMAD4	SMAD family member 4	Transcription factor	4089
739	CAMK2N1	calcium/calmodulin dependent protein kinase II inhibitor 1		55450
740	PLK4	polo like kinase 4	Kinase	10733
741	MGMT	O-6-methylguanine-DNA methyltransferase		4255
742	SHMT1	serine hydroxymethyltransferase 1	Enzyme	6470
743	TRIP13	thyroid hormone receptor interactor 13		9319
744	IL6	interleukin 6		3569
745	PTBP1	polypyrimidine tract binding protein 1		5725
746	LPAR2	lysophosphatidic acid receptor 2	G-protein coupled receptor	9170
747	STAG3	stromal antigen 3	Nucleic acid binding	10734
748	VDR	vitamin D receptor	Nuclear receptor	7421
749	ACE	angiotensin I converting enzyme	Enzyme	1636
750	L1CAM	L1 cell adhesion molecule		3897
751	FASLG	Fas ligand	Signaling	356
752	RGS5	regulator of G protein signaling 5	Enzyme modulator	8490
753	MIR330	microRNA 330		442902
754	ABCG2	ATP binding cassette subfamily G member 2 (Junior blood group)	Transporter	9429
755	FUT1	fucosyltransferase 1 (H blood group)	Enzyme	2523
756	MIR205	microRNA 205		406988
757	CHP2	calcineurin like EF-hand protein 2		63928
758	CD59	CD59 molecule (CD59 blood group)		966
759	MIR337	microRNA 337		442905
760	MIR655	microRNA 655		724025
761	MIR655	microRNA 655		724025
762	PALB2	partner and localizer of BRCA2		79728
763	PIK3CG	phosphatidylinositol-4,5-bisphosphate 3-kinase catalytic subunit gamma	Kinase	5294
764	MIR23B	microRNA 23b		407011
765	MIR200A	microRNA 200a		406983
766	PIK3CA	phosphatidylinositol-4,5-bisphosphate 3-kinase catalytic subunit alpha	Kinase	5290
767	ERBB3	erb-b2 receptor tyrosine kinase 3	Kinase	2065
768	SDC1	syndecan 1	Extracellular structure	6382
769	XPO1	exportin 1	Receptor	7514
770	MIR142	microRNA 142		406934
771	CLU	clusterin		1191
772	CXCL12	C-X-C motif chemokine ligand 12		6387
773	TOP2A	DNA topoisomerase II alpha		7153
774	CX3CL1	C-X3-C motif chemokine ligand 1	Signaling	6376
775	FEN1	flap structure-specific endonuclease 1	Enzyme	2237
776	IGF1R	insulin like growth factor 1 receptor	Kinase	3480
777	ALDH1A1	aldehyde dehydrogenase 1 family member A1	Enzyme	216

778	SPARCL1	SPARC like 1	Signaling	8404
779	CARTPT	CART prepropeptide		9607
780	TNFRSF10B	TNF receptor superfamily member 10b		8795
781	HIF1A	hypoxia inducible factor 1 subunit alpha	Transcription factor	3091
782	RELA	RELA proto-oncogene, NF-kB subunit	Transcription factor	5970
783	ST14	suppression of tumorigenicity 14	Enzyme	6768
784	SURF6	surfeit 6	Nucleic acid binding	6838
785	MLLT10	MLLT10 histone lysine methyltransferase DOT1L cofactor	Transcription factor	8028
786	S100A10	S100 calcium binding protein A10	Calcium-binding protein	6281
787	ACTB	actin beta	Cellular structure	60
788	TLR4	toll like receptor 4		7099
789	MIR17	microRNA 17		406952
790	INSR	insulin receptor	Kinase	3643
791	PHF13	PHD finger protein 13	Epigenetic regulator	148479
792	FGF3	fibroblast growth factor 3	Signaling	2248
793	MIR383	microRNA 383		494332
794	CT45A1	cancer/testis antigen family 45 member A1		541466
795	CHD5	chromodomain helicase DNA binding protein 5		26038
796	FLOT1	flotillin 1		10211
797	AKT1	AKT serine/threonine kinase 1	Kinase	207
798	MIR130B	microRNA 130b		406920
799	HSD17B12	hydroxysteroid 17-beta dehydrogenase 12		51144
800	NES	nestin		10763
801	MALAT1	metastasis associated lung adenocarcinoma transcript 1		378938
802	MAP2K1	mitogen-activated protein kinase kinase 1	Kinase	5604
803	MIR874	microRNA 874		1E+08
804	MIR217	microRNA 217		406999
805	WNT4	Wnt family member 4	Signaling	54361
806	MIR520G	microRNA 520g		574484
807	AKR1C3	aldo-keto reductase family 1 member C3	Enzyme	8644
808	SPARC	secreted protein acidic and cysteine rich	Signaling	6678
809	SERPINC1	serpin family C member 1	Enzyme modulator	462
810	F3	coagulation factor III, tissue factor	Receptor	2152
811	NR1I2	nuclear receptor subfamily 1 group I member 2	Nuclear receptor	8856
812	KPNB1	karyopherin subunit beta 1	Transporter	3837
813	FOXO3	forkhead box O3		2309
814	KHDRBS1	KH RNA binding domain containing, signal transduction associated 1	Nucleic acid binding	10657
815	CTHRC1	collagen triple helix repeat containing 1	Enzyme	115908
816	GPC6	glypican 6		10082
817	CDKN2A	cyclin dependent kinase inhibitor 2A		1029
818	CASP2	caspase 2	Enzyme	835
819	SERBP1	SERPINE1 mRNA binding protein 1	Nucleic acid binding	26135

820	CSF1R	colony stimulating factor 1 receptor	Kinase	1436
821	ATAD5	ATPase family AAA domain containing 5		79915
822	AR	androgen receptor	Nuclear receptor	367
823	PLXDC1	plexin domain containing 1		57125
824	PNPO	pyridoxamine 5'-phosphate oxidase	Enzyme	55163
825	CDH5	cadherin 5		1003
826	SSTR4	somatostatin receptor 4	G-protein coupled receptor	6754
827	FOSL1	FOS like 1, AP-1 transcription factor subunit	Transcription factor	8061
828	GSTT1	glutathione S-transferase theta 1		2952
829	CD40LG	CD40 ligand	Signaling	959
830	ARHGAP27	Rho GTPase activating protein 27	Enzyme modulator	201176
831	PRSS1	serine protease 1	Enzyme	5644
832	MIR551B	microRNA 551b		693136
833	SRY	sex determining region Y	Transcription factor	6736
834	ADAM12	ADAM metallopeptidase domain 12	Enzyme	8038
835	GAS5	growth arrest specific 5		60674
836	B4GALNT1	beta-1,4-N-acetyl-galactosaminyltransferase 1		2583
837	SMO	smoothened, frizzled class receptor	G-protein coupled receptor	6608
838	UTRN	utrophin		7402
839	RHOC	ras homolog family member C	Enzyme modulator	389
840	MKNK1	MAPK interacting serine/threonine kinase 1	Kinase	8569
841	MSH6	mutS homolog 6	Nucleic acid binding	2956
842	FOXA2	forkhead box A2	Transcription factor	3170
843	PTK2	protein tyrosine kinase 2	Kinase	5747
844	MIR34B	microRNA 34b		407041
845	ALCAM	activated leukocyte cell adhesion molecule	Cell adhesion	214
846	EZR	ezrin	Cellular structure	7430
847	FGFR3	fibroblast growth factor receptor 3	Kinase	2261
848	TP73-AS1	TP73 antisense RNA 1		57212
849	CHI3L1	chitinase 3 like 1		1116
850	VTCN1	V-set domain containing T cell activation inhibitor 1	Enzyme modulator	79679
851	KIF2A	kinesin family member 2A	Cellular structure	3796
852	RARB	retinoic acid receptor beta	Nuclear receptor	5915
853	TCF4	transcription factor 4	Transcription factor	6925
854	AURKA	aurora kinase A	Kinase	6790
855	AHCYL1	adenosylhomocysteinase like 1	Enzyme	10768
856	KRT17	keratin 17		3872
857	TRAF2	TNF receptor associated factor 2	Signaling	7186
858	MIR29B1	microRNA 29b-1		407024
859	SNAI1	snail family transcriptional repressor 1		6615
860	CHAF1A	chromatin assembly factor 1 subunit A	Nucleic acid binding	10036
861	GC	GC vitamin D binding protein	Transporter	2638
862	MIR424	microRNA 424		494336

863	FRA7G	fragile site, aphidicolin type, common, fra(7)(q31.2)		2414
864	FANCA	FA complementation group A		2175
865	EGF	epidermal growth factor		1950
866	USP17L2	ubiquitin specific peptidase 17 like family member 2		377630
867	THY1	Thy-1 cell surface antigen		7070
868	KIT	KIT proto-oncogene, receptor tyrosine kinase	Kinase	3815
869	MIR210	microRNA 210		406992
870	WNT5A	Wnt family member 5A	Signaling	7474
871	KLK4	kallikrein related peptidase 4	Enzyme	9622
872	IL31	interleukin 31		386653
873	CTNNB1	catenin beta 1		1499
874	MIR141	microRNA 141		406933
875	FSHR	follicle stimulating hormone receptor	G-protein coupled receptor	2492
876	BAG3	BAG cochaperone 3		9531
877	CCND1	cyclin D1	Enzyme modulator	595
878	DKK4	dickkopf WNT signaling pathway inhibitor 4		27121
879	SIK2	salt inducible kinase 2	Kinase	23235
880	RPL17	ribosomal protein L17		6139
881	BCL6	BCL6 transcription repressor		604
882	RAB25	RAB25, member RAS oncogene family		57111
883	TGFB1I1	transforming growth factor beta 1 induced transcript 1	Cellular structure	7041
884	LMX1A	LIM homeobox transcription factor 1 alpha	Nucleic acid binding	4009
885	ABCA12	ATP binding cassette subfamily A member 12	Transporter	26154
886	MRC1	mannose receptor C-type 1		4360
887	CLIC1	chloride intracellular channel 1	Ion channel	1192
888	SLC2A1	solute carrier family 2 member 1	Transporter	6513
889	EPCAM	epithelial cell adhesion molecule		4072
890	HAGLR	HOXD antisense growth-associated long non-coding RNA		401022
891	CDH3	cadherin 3		1001
892	KDM5B	lysine demethylase 5B	Epigenetic regulator	10765
893	PRR13	proline rich 13		54458
894	FLT1	fms related receptor tyrosine kinase 1	Kinase	2321
895	HOTAIR	HOX transcript antisense RNA		1E+08
896	SMAD2	SMAD family member 2	Transcription factor	4087
897	GSTM1	glutathione S-transferase mu 1		2944
898	PRSS2	serine protease 2	Enzyme	5645
899	PGF	placental growth factor	Signaling	5228
900	MIR214	microRNA 214		406996
901	DKK3	dickkopf WNT signaling pathway inhibitor 3		27122
902	POSTN	periostin	Signaling	10631
903	PLA2G1B	phospholipase A2 group IB	Enzyme	5319

904	TCEAL7	transcription elongation factor A like 7	Transcription factor	56849
905	ICMT	isoprenylcysteine carboxyl methyltransferase		23463
906	ANIB1	aneurysm, intracranial berry 1		116833
907	PRIMA1	proline rich membrane anchor 1		145270
908	AKAP6	A-kinase anchoring protein 6		9472
909	PRPF31	pre-mRNA processing factor 31	Nucleic acid binding	26121
910	NAT2	N-acetyltransferase 2	Enzyme	10
911	XIST	X inactive specific transcript		7503
912	F2R	coagulation factor II thrombin receptor	G-protein coupled receptor	2149
913	MIR143	microRNA 143		406935
914	MAP2K7	mitogen-activated protein kinase kinase 7	Kinase	5609
915	AFDN	afadin, adherens junction formation factor	Cellular structure	4301
916	STIP1	stress induced phosphoprotein 1		10963
917	HLA-G	major histocompatibility complex, class I, G		3135
918	MTDH	metadherin		92140
919	TRPC6	transient receptor potential cation channel subfamily C member 6	Ion channel	7225
920	FBP1	fructose-bisphosphatase 1	Enzyme	2203
921	TERT	telomerase reverse transcriptase	Enzyme	7015
922	SPA17	sperm autoantigenic protein 17		53340
923	GALNT14	polypeptide N-acetylgalactosaminyltransferase 14	Enzyme	79623
924	FBXW7	F-box and WD repeat domain containing 7	Enzyme modulator	55294
925	KRT7	keratin 7		3855
926	STS	steroid sulfatase	Enzyme	412
927	TP53	tumor protein p53	Transcription factor	7157
928	ABHD11-AS1	ABHD11 antisense RNA 1 (tail to tail)		171022
929	STK11	serine/threonine kinase 11	Kinase	6794
930	MIR184	microRNA 184		406960
931	HNF1B	HNF1 homeobox B	Nucleic acid binding	6928
932	CTAG1B	cancer/testis antigen 1B		1485
933	ADAM17	ADAM metallopeptidase domain 17		6868
934	LINC00968	long intergenic non-protein coding RNA 968		1.01E+08
935	USP5	ubiquitin specific peptidase 5	Enzyme	8078
936	CFLAR	CASP8 and FADD like apoptosis regulator	Enzyme	8837
937	MIR150	microRNA 150		406942
938	SPRY4	sprouty RTK signaling antagonist 4	Signaling	81848
939	MXD1	MAX dimerization protein 1	Transcription factor	4084
940	SIRT1	sirtuin 1	Epigenetic regulator	23411
941	TNFRSF6B	TNF receptor superfamily member 6b		8771
942	UGT1A	UDP glucuronosyltransferase family 1 member A complex locus		7361
943	IL1B	interleukin 1 beta		3553
944	BRS3	bombesin receptor subtype 3	G-protein coupled	680

			receptor	
945	DNM1L	dynamin 1 like	Enzyme modulator	10059
946	MIR381	microRNA 381		494330
947	GAPDH	glyceraldehyde-3-phosphate dehydrogenase	Enzyme	2597
948	APC	APC regulator of WNT signaling pathway		324
949	LINC00673	long intergenic non-protein coding RNA 673		1E+08
950	GPX1	glutathione peroxidase 1	Enzyme	2876
951	GNA12	G protein subunit alpha 12	Enzyme modulator	2768
952	LIMK1	LIM domain kinase 1	Kinase	3984
953	BCL10	BCL10 immune signaling adaptor		8915
954	TEX11	testis expressed 11		56159
955	ESR2	estrogen receptor 2	Nuclear receptor	2100
956	HGF	hepatocyte growth factor	Enzyme	3082
957	PPM1D	protein phosphatase, Mg2+/Mn2+ dependent 1D	Enzyme	8493
958	CYP1B1	cytochrome P450 family 1 subfamily B member 1	Enzyme	1545
959	ROS1	ROS proto-oncogene 1, receptor tyrosine kinase	Kinase	6098
960	PVR	PVR cell adhesion molecule		5817
961	DHCR24	24-dehydrocholesterol reductase	Enzyme	1718
962	PLAU	plasminogen activator, urokinase	Enzyme	5328
963	PTEN	phosphatase and tensin homolog	Enzyme	5728
964	MIR328	microRNA 328		442901
965	SERPINB5	serpin family B member 5	Enzyme modulator	5268
966	NGF	nerve growth factor	Signaling	4803
967	NANOG	Nanog homeobox	Transcription factor	79923
968	TGM2	transglutaminase 2	Enzyme	7052
969	PTOV1	PTOV1 extended AT-hook containing adaptor protein	Transcription factor	53635
970	PTPN11	protein tyrosine phosphatase non-receptor type 11		5781
971	PLK1	polo like kinase 1	Kinase	5347
972	LGALS1	galectin 1	Signaling	3956
973	COL15A1	collagen type XV alpha 1 chain		1306
974	CDKN1B	cyclin dependent kinase inhibitor 1B		1027
975	BTRC	beta-transducin repeat containing E3 ubiquitin protein ligase		8945
976	JTB	jumping translocation breakpoint		10899
977	MUC1	mucin 1, cell surface associated		4582
978	MYH9	myosin heavy chain 9		4627
979	MIR196B	microRNA 196b		442920
980	DCLK1	doublecortin like kinase 1	Kinase	9201
981	HOXB3	homeobox B3		3213
982	CASP8	caspase 8	Enzyme	841
983	MIR203A	microRNA 203a		406986
984	CCNG2	cyclin G2	Enzyme modulator	901
985	MIR137	microRNA 137		406928
986	MIR494	microRNA 494		574452

987	SLC1A5	solute carrier family 1 member 5	Transporter	6510
988	CXCL11	C-X-C motif chemokine ligand 11	Signaling	6373
989	NRAS	NRAS proto-oncogene, GTPase	Enzyme modulator	4893
990	OGG1	8-oxoguanine DNA glycosylase		4968
991	PIK3R3	phosphoinositide-3-kinase regulatory subunit 3	Enzyme modulator	8503
992	MTOR	mechanistic target of rapamycin kinase	Kinase	2475
993	WT1	WT1 transcription factor	Nucleic acid binding	7490
994	LIMA1	LIM domain and actin binding 1	Enzyme	51474
995	HSPB2	heat shock protein family B (small) member 2		3316
996	KLRK1	killer cell lectin like receptor K1		22914
997	RAD51-AS1	RAD51 antisense RNA 1		1.01E+08
998	SOX3	SRY-box transcription factor 3	Transcription factor	6658
999	CALR	calreticulin	Calcium-binding protein	811
1000	RAD52	RAD52 homolog, DNA repair protein	Nucleic acid binding	5893
1001	RSPO1	R-spondin 1		284654
1002	SMN2	survival of motor neuron 2, centromeric		6607
1003	BABAM1	BRISC and BRCA1 A complex member 1		29086
1004	SEPTIN9	septin 9	Enzyme modulator	10801
1005	PRDX5	peroxiredoxin 5		25824
1006	CD46	CD46 molecule		4179
1007	CCK	cholecystokinin		885
1008	TUBB3	tubulin beta 3 class III	Cellular structure	10381
1009	S100A6	S100 calcium binding protein A6	Calcium-binding protein	6277
1010	MIAT	myocardial infarction associated transcript		440823
1011	ADRM1	adhesion regulating molecule 1		11047
1012	DDX4	DEAD-box helicase 4		54514
1013	AIMP2	aminoacyl tRNA synthetase complex interacting multifunctional protein 2		7965
1014	ARNTL	aryl hydrocarbon receptor nuclear translocator like	Transcription factor	406
1015	CTCF	CCCTC-binding factor	Transcription factor	10664
1016	CCL2	C-C motif chemokine ligand 2	Signaling	6347
1017	ANGPT1	angiopoietin 1	Signaling	284
1018	HMGA2	high mobility group AT-hook 2	Nucleic acid binding	8091
1019	CREB5	cAMP responsive element binding protein 5	Transcription factor	9586
1020	ANGPT2	angiopoietin 2	Signaling	285
1021	CLOCK	clock circadian regulator		9575
1022	KDR	kinase insert domain receptor	Kinase	3791
1023	TNC	tenascin C		3371
1024	MARCKS	myristoylated alanine rich protein kinase C substrate		4082
1025	MIR145	microRNA 145		406937
1026	PLK2	polo like kinase 2	Kinase	10769
1027	IGF2	insulin like growth factor 2		3481

1028	BRIP1	BRCA1 interacting protein C-terminal helicase 1	Enzyme	83990
1029	KLK13	kallikrein related peptidase 13	Enzyme	26085
1030	IL2	interleukin 2		3558
1031	CDH1	cadherin 1		999
1032	SERPINB6	serpin family B member 6	Enzyme modulator	5269
1033	IL36A	interleukin 36 alpha		27179
1034	PLEKHM1	pleckstrin homology and RUN domain containing M1		9842
1035	MSX1	msh homeobox 1	Transcription factor	4487
1036	EGFL6	EGF like domain multiple 6		25975
1037	SLC9A1	solute carrier family 9 member A1	Transporter	6548
1038	CTSL	cathepsin L	Enzyme	1514
1039	MIR100	microRNA 100		406892
1040	HOXA4	homeobox A4		3201
1041	OGFR	opioid growth factor receptor	Receptor	11054
1042	TGFA	transforming growth factor alpha	Signaling	7039
1043	KIF15	kinesin family member 15	Cellular structure	56992
1044	HSF1	heat shock transcription factor 1	Transcription factor	3297
1045	HOXA9	homeobox A9		3205
1046	ITM2A	integral membrane protein 2A		9452
1047	NFKBIB	NFKB inhibitor beta		4793
1048	SLC46A1	solute carrier family 46 member 1	Transporter	113235
1049	MIR139	microRNA 139		406931
1050	ABHD11	abhydrolase domain containing 11		83451
1051	VCAM1	vascular cell adhesion molecule 1		7412
1052	REL	REL proto-oncogene, NF-kB subunit	Transcription factor	5966
1053	MIR595	microRNA 595		693180
1054	RAD51D	RAD51 paralog D		5892
1055	MIR146B	microRNA 146b		574447
1056	FOXP1	forkhead box P1		27086
1057	CPS1-IT1	CPS1 intronic transcript 1		29034
1058	C4BPB	complement component 4 binding protein beta		725
1059	SOX11	SRY-box transcription factor 11	Transcription factor	6664
1060	CCN3	cellular communication network factor 3	Signaling	4856
1061	F11R	F11 receptor		50848
1062	TXN	thioredoxin		7295
1063	DDR1	discoidin domain receptor tyrosine kinase 1	Kinase	780
1064	PTGS1	prostaglandin-endoperoxide synthase 1	Enzyme	5742
1065	HOXC6	homeobox C6	Transcription factor	3223
1066	MIR1307	microRNA 1307		1E+08
1067	PVT1	Pvt1 oncogene		5820
1068	ANXA4	annexin A4		307
1069	MIR186	microRNA 186		406962
1070	GAB1	GRB2 associated binding protein 1	Receptor	2549
1071	BAX	BCL2 associated X, apoptosis regulator	Signaling	581
1072	GORASP1	golgi reassembly stacking protein 1		64689

1073	MIR212	microRNA 212		406994
1074	DACT1	dishevelled binding antagonist of beta catenin 1		51339
1075	ERCC2	ERCC excision repair 2, TFIIH core complex helicase subunit	Enzyme	2068
1076	CSF2	colony stimulating factor 2	Signaling	1437
1077	MMP9	matrix metallopeptidase 9	Enzyme	4318

Table S3. Potential targets of *C. fistula* for EOC./

Sr. No.	Proteins	Gene	Uniprot ID
01	Serine Protease 1	PRSS1	P07477
02	Myeloperoxidase	MPO	P05164
03	Insulin Like Growth Factor 1 Receptor	IGF1R	P08069
04	Steroid Sulfatase	STS	P08842
05	Plasminogen Activator, Urokinase	PLAU	P00749
06	Checkpoint Kinase 2	CHEK2	O96017
07	Kinase Insert Domain Receptor	KDR	P35968
08	Cytochrome P450 Family-1 Subfamily-B Member-1	CYP1B1	Q16678
09	AKT Serine/Threonine Kinase 1	AKT1	P31749
10	Insulin Like Growth Factor Binding Protein 3	IGFBP3	P17936
11	Coagulation Factor-II Thrombin Receptor	F2R	P25116
12	Casein Kinase 2 Alpha 2	CSNK2A2	P19784
13	Androgen Receptor	AR	P10275
14	ATP Citrate Lyase	ACLY	P53396
15	Maternal Embryonic Leucine Zipper Kinase	MELK	Q14680
16	Angiotensin II Receptor Type 1	AGTR1	P30556
17	Progesterone Receptor	PGR	P06401
18	ATP-Binding Cassette Subfamily-G Member-2	ABCG2	Q9UNQ0
19	Indoleamine 2,3-Dioxygenase 1	IDO1	P14902
20	Colony Stimulating Factor 1 Receptor	CSF1R	P07333
21	ATP Binding Cassette Subfamily B Member 1	ABCB1	P08183
22	Arachidonate 15-Lipoxygenase	ALOX15	P16050

23	Cytochrome-P450 Family-19 Subfamily-A Member-1	CYP19A1	P11511
24	Telomerase Reverse Transcriptase	TERT	O14746
25	Elastase, Neutrophil Expressed	ELANE	P08246
26	Chemokine Receptor 1 C-C Motif	CCR1	P32246
27	Polo Like Kinase 4	PLK4	O00444
28	Estrogen Related Receptor Alpha	ESRRA	P11474
29	Glutathione S-Transferase Mu 1	GSTM1	P09488
30	Angiotensin I Converting Enzyme	ACE	P12821
31	Vitamin D Receptor	VDR	P11473
32	Insulin Receptor	INSR	P06213
33	Polo Like Kinase 1	PLK1	P53350
34	B Raf Proto-Oncogene, Serine-Threonine Kinase	BRAF	P15056
35	Estrogen Receptor 1	ESR1	P03372
36	Protein Tyrosine Kinase-2	PTK2	Q05397
37	Interleukin 2	IL2	P60568
38	Estrogen Receptor 2	ESR2	Q92731
39	Nuclear Factor Kappa B Subunit 1	NFKB1	P19838
40	Cyclin-Dependent Kinase 6	CDK6	Q00534
41	Cyclin-Dependent Kinase 4	CDK4	P11802
42	Cyclin-Dependent Kinase 2	CDK2	P24941
43	Albumin	ALB	P02768
44	BCL2 Apoptosis Regulator	BCL2	P10415
45	MDM2 Proto-Oncogene	MDM2	Q00987
46	Cyclin-Dependent Kinase 1	CDK1	P06493
47	Peptidylprolyl Cis And Trans Isomerase, NIMA-Interacting 1	PIN1	Q13526
48	Sex-Hormone Binding Globulin	SHBG	P04278
49	BCL2 Like 1	BCL2L1	Q07817
50	DNA Topoisomerase-II Alpha	TOP2A	P11388
51	Lysine Demethylase 5B	KDM5B	Q9UGL1
52	Phosphatidylinositol 4, 5-Bisphosphate 3-Kinase Catalytic Subunit (Gamma)	PIK3CG	P48736

53	Inhibitor Of Nuclear Factor Kappa B Kinase Subunit Beta(IKBKB)	IKBKB	O14920
54	Caspase 8	CASP8	Q14790
55	Caspase 3	CASP3	P42574
56	Caspase 2	CASP2	P42575
57	Carbonic Anhydrase 9	CA9	Q16790
58	Heat-Shock Protein 90 Family (Alpha) Class A Member 1	HSP90AA1	P07900
59	Poly ADP Ribose Polymerase 1	PARP1	P09874
60	Matrix-Metallopeptidase 1	MMP1	P03956
61	Matrix-Metallopeptidase 2	MMP2	P08253
62	Matrix-Metallopeptidase 3	MMP3	P08254
63	Matrix-Metallopeptidase 9	MMP9	P14780
64	Sirtuin 1	SIRT1	Q96EB6
65	Matrix-Metallopeptidase 14	MMP14	P50281
66	Phosphatidylinositol-4,5-Bisphosphate 3-Kinase Catalytic Subunit Alpha	PIK3CA	P42336
67	Interleukin 1 Beta	IL1B	P01584
68	KIT Proto-Oncogene, Receptor Tyrosine Kinase	KIT	P10721
69	Microtubule Associated Protein Tau	MAPT	P10636
70	Fructose-Bisphosphatase 1	FBP1	P09467
71	MET Proto-Oncogene, Receptor Tyrosine Kinase	MET	P08581
72	Phospholipase A2 Group IB	PLA2G1B	P04054
73	Paraoxonase 1	PON1	P27169
74	Prostaglandin-Endoperoxide Synthase 2	PTGS2	P35354
75	Thymidylate Synthetase	TYMS	P04818
76	RELA Proto-Oncogene, NF-Kb Subunit	RELA	Q04206
77	Epidermal Growth Factor Receptor	EGFR	P00533
78	Prostaglandin-Endoperoxide Synthase 1	PTGS1	P23219
79	Cytochrome P450 Family 17 Subfamily A Member 1	CYP17A1	P05093
80	Aurora Kinase A	AURKA	O14965
81	Mitogen Activated Protein Kinase 8	MAPK8	P45983

82	Erb-B2 Receptor Tyrosine Kinase 2	ERBB2	P04626
83	KISS1 Receptor	KISS1R	Q969F8
84	Solute Carrier Family 13 Member 5	SLC13A5	Q86YT5
85	Mitogen-Activated Protein Kinase 3	MAPK3	P27361
86	Synuclein Alpha	SNCA	P37840
87	NADPH Quinone Dehydrogenase 1	NQO1	P15559
88	Coagulation Factor X	F10	P00742
89	Nuclear Receptor Subfamily 1 Group H Member 2	NR1H2	P55055
90	LIM Domain Kinase 1	LIMK1	P53667
91	Aldo Keto Reductase Family 1 Member C3	AKR1C3	P42330
92	Protein Tyrosine Phosphatase Non-Receptor Type 11	PTPN11	Q06124
93	Mitogen-Activated Protein Kinase 14	MAPK14	Q16539
94	Programmed Cell Death 4	PDCD4	Q53EL6
95	Catenin Beta-1	CTNNB1	P35222
96	Protein Tyrosine Phosphatase Non-Receptor Type 6	PTPN6	P29350
97	TEK Receptor Tyrosine Kinase	TEK	Q02763
98	Heat Shock Protein Family A (Hsp70) Member 1A	HSPA1A	P0DMV8
